# Supplementary material for: REPROGRAM: REsilience PROmotion with GeRoprotectors: AssessMent of biological effect: Rationale and protocol for a trial of biological effect
Source: PLoS One. 2026 Jun 17;21(6):e0346347. doi: 10.1371/journal.pone.0346347 (PMC13274875; doi:10.1371/journal.pone.0346347)
Supplement: S2 File — (DOCX) [file pone.0346347.s002.docx]

| ​​​UNIVERSITY OF BIRMINGHAM ​ | | | |
| --- | --- | --- | --- |
| **​​REsilience PROmotion with GeRoprotectors: AssessMent of biological effect** | | | |
| ​​REPROGRAM Study | | | |
| ​  REPROGRAM  ​  ​  ​  ​  ​ | | | |
| **​​Version 5.0** | | | |
| **​21/01/2026** | | | |
| **​IRAS Number** | ​ 338321 |  |  |
| **​Sponsor reference number** | ​ RG_24-024 |  |  |
| **​REC reference number** | ​ |  |  |

​This protocol has regard for the HRA guidance

​

Signature page

The undersigned confirm that the following protocol has been agreed and accepted and that the Chief Investigator agrees to adhere to the signed University of Birmingham’s Sponsorship CI declaration.

I agree to ensure that the confidential information contained in this document will not be used for any other purpose other than the evaluation or conduct of the investigation without the prior written consent of the Sponsor

I also confirm that I will make the findings of the study publicly available through publication or other dissemination tools without any unnecessary delay and that an honest accurate and transparent account of the study will be given; and that any discrepancies from the study as planned in this protocol will be explained.

Chief Investigator:

Signature: Date:

Name:

Sponsor statement:

The University of Birmingham has taken on the sponsor role for protocol development oversight; the signing of the IRAS form by the sponsor will serve as confirmation of approval of this protocol.

**​**

Contents

[1. Background 12](#_Toc215751840)

[2. Rationale 14](#_Toc215751841)

[Patient and public involvement 14](#_Toc215751842)

[Rationale for research questions – relevance to NHS patients and healthcare policy 14](#_Toc215751843)

[Rationale for study design 14](#_Toc215751844)

[3. Research question/aims 15](#_Toc215751845)

[Objectives 15](#_Toc215751846)

[Outcomes 15](#_Toc215751847)

[4. Study design and methods of data collection and data analysis 16](#_Toc215751848)

[Power Calculations 16](#_Toc215751849)

[Data management 16](#_Toc215751850)

[Data analysis 16](#_Toc215751851)

[Data Protection 17](#_Toc215751852)

[GP Notification 17](#_Toc215751853)

[Intervention 17](#_Toc215751854)

[5. Study setting 19](#_Toc215751855)

[6. Participant Recruitment 21](#_Toc215751856)

[Eligibility Criteria 21](#_Toc215751857)

[Inclusion criteria 21](#_Toc215751858)

[Exclusion criteria 21](#_Toc215751859)

[Recruitment target 22](#_Toc215751860)

[Recruitment technique 22](#_Toc215751861)

[Recruitment 22](#_Toc215751862)

[Consent 22](#_Toc215751863)

[7. Study procedures 24](#_Toc215751864)

[Co-morbidities and medications 25](#_Toc215751865)

[Handgrip strength 25](#_Toc215751866)

[Short physical performance battery 25](#_Toc215751867)

[Activities of Daily Living (ADL) assessment 25](#_Toc215751868)

[Frailty assessments 26](#_Toc215751869)

[Cognitive testing 26](#_Toc215751870)

[Stool sample collection 26](#_Toc215751871)

[Venepuncture 27](#_Toc215751872)

[Adipose biopsies 27](#_Toc215751873)

[Ultrasound quadriceps 27](#_Toc215751874)

[Bioelectrical Impedance Analysis 28](#_Toc215751875)

[Three-day food record 28](#_Toc215751876)

[Adverse Event Reporting 28](#_Toc215751877)

[Drug Distribution 29](#_Toc215751878)

[Compliancy Pill Check 29](#_Toc215751879)

[**8.** **REsilience and Frailty Research – Lived Experience of Community-dwelling Older AdulTs** 30](#_Toc215751880)

[8.1 Background 30](#_Toc215751881)

[8.2 Aims 30](#_Toc215751882)

[8.3 Design and setting 30](#_Toc215751883)

[8.4 Participant identification, recruitment and consent 30](#_Toc215751884)

[8.5 Data collection 31](#_Toc215751885)

[8.6 Sample size and information power 31](#_Toc215751886)

[8.7 Data management and protection 32](#_Toc215751887)

[8.8 Data analysis 32](#_Toc215751888)

[8.9 Outputs, dissemination and integration with the main trial 32](#_Toc215751889)

[9. Detailed laboratory methodology for mechanistic studies 33](#_Toc215751890)

[Stool microbiome and metagenomics 33](#_Toc215751891)

[DNA methylation, immune system ageing, compromised autophagy, mitochondrial function, nutrient sensing and stem cell exhaustion 33](#_Toc215751892)

[Adipose tissue to assess cellular senescence and tissue-based resilience biomarkers 33](#_Toc215751893)

[Inflammatory and senescence markers 34](#_Toc215751894)

[10. Safety reporting 34](#_Toc215751895)

[11. Ethical and regulatory considerations 35](#_Toc215751896)

[Assessment and management of risk 36](#_Toc215751897)

[Research Ethics Committee (REC) and other Regulatory review & reports 38](#_Toc215751898)

[Regulatory Review & Compliance 39](#_Toc215751899)

[Amendments 39](#_Toc215751900)

[Patient & Public Involvement 39](#_Toc215751901)

[Protocol compliance 39](#_Toc215751902)

[Data protection and patient confidentiality 40](#_Toc215751903)

[Storage of Samples 40](#_Toc215751904)

[Indemnity 40](#_Toc215751905)

[End of study and archiving 41](#_Toc215751906)

[Access to the final study dataset 41](#_Toc215751907)

[12. Dissemination 42](#_Toc215751908)

[Dissemination policy 42](#_Toc215751909)

[Authorship eligibility guidelines and any intended use of professional writers 42](#_Toc215751910)

[13. References 43](#_Toc215751911)

[Appendix 46](#_Toc215751912)

[Criteria for sarcopenia diagnosis 46](#_Toc215751913)

[Short Physical Performance Battery (SPPB) 47](#_Toc215751914)

[Barthel Index 48](#_Toc215751915)

[Nottingham Extended Activities of Daily Living 49](#_Toc215751916)

[Criteria used to make a phenotypic diagnosis of frailty 50](#_Toc215751917)

[Scoring criteria for CFS 51](#_Toc215751918)

[Frailty Index 52](#_Toc215751919)

Abbreviations

ADL Activities of daily living

ADSC Adipocyte derived stem cells

AE Adverse event

AR Adverse reaction

BATT Bilateral anterior thigh thickness

BIA Bioelectrical impedance analysis

CI Chief Investigator

CRF Case Report Form

CPRD Clinical Practice Research Database

DPA Data protection committee

EEG Electroencephalogram

ELISA Enzyme-linked immunosorbent assay

GCP Good Clinical Practice

GP General Practitioner

HRA Health Research Authority

ICF Informed Consent Form

mTOR Mammalian target of Rapamycin

NHS National Health Service

PI Principal Investigator

PIL Participant/ Patient Information Leaflet

R&D NHS Trust R&D Department

REC Research Ethics Committee

SAE Serious Adverse Event

SAR Serious Adverse Reaction

SA-β-GAL Senescence-associated beta-galactosidase

SMF Study Master File

SOP Standard Operating Procedure

SPPB Short physical performance battery

SUSAR Suspected Unexpected Serious Adverse Reactions

TAF Thiosulfation aldehyde fuchsin

UAR Unexplained Adverse Reaction

Key study contacts

| **Sponsor** | |
| --- | --- |
| University of Birmingham |  |
| Sponsor contact: Dr Birgit Whitman  Head of Research Governance and Integrity | [researchgovernance@contacts.bham.ac.uk](mailto:researchgovernance@contacts.bham.ac.uk) |

| **Chief investigator** | |
| --- | --- |
| Dr Daisy Wilson | Clinical Lecturer in Academic Geriatric Medicine and Honorary Consultant Geriatrician |
| Institute of Inflammation and Ageing  University of Birmingham  Birmingham  B15 2TT | 0121 371 3264  d.v.wilson@bham.ac.uk |

| **Other principal investigators** | |
| --- | --- |
| Dr Thomas Jackson  Institute of Inflammation and Ageing  University of Birmingham  Birmingham  B15 2TT | Lead Investigator for Wellcome LEAP grant  Associate Professor of Ageing and Honorary Consultant Geriatrician  0121 371 3264  [t.jackson@bham.ac.uk](mailto:t.jackson@bham.ac.uk) |
| Dr Carly Welch  Department of Twins Research  King’s College London  3^rd^ Floor, St Thomas’ Hospital  Westminster Bridge  London  SE1 7EH | Lead investigator at King’s College London  Principal Investigator at Guy’s and St Thomas’ Hospital NHS Foundation Trust    Consultant Geriatrician and Senior Clinical Lecturer    020 7188 7188  [carly.welch@kcl.ac.uk](mailto:carly.welch@kcl.ac.uk) |
| Dr Helen McGettrick  Institute of Inflammation and Ageing  University of Birmingham  Birmingham  B15 2TT | Lead investigator for experimental biology    Reader in Inflammation and Vascular biology    0121 371 3228  [h.m.mcgettrick@bham.ac.uk](mailto:h.m.mcgettrick@bham.ac.uk) |

Funding and support in kind

| **FUNDER(S)** | **FINANCIAL AND NON-FINANCIAL SUPPORT GIVEN** |
| --- | --- |
| Wellcome LEAP Dynamic Resilience programme | Financial support to cover direct and indirect research costs. The Dynamic Resilience programme is an ambitious global programme that promotes early collaboration between grant recipients. The funder will meet regularly with grant recipients throughout the course of funding, which includes this study. |

Role of study sponsor and funder

The study sponsor and the funder will not be involved in the study design, conduct, data analysis and interpretation, manuscript writing, or dissemination of results. The funder will not control the final decision regarding any of these aspects of the study. The sponsor has approved the final version of the protocol to ensure that it is compatible with University of Birmingham policy and Standard Operating Procedures and is responsible for the oversight of the study.

Roles and responsibilities of study management committees/groups and individuals

**Chief investigator**

Dr Daisy Wilson will act as the Chief Investigator (CI) for the duration of this study. The CI will be responsible for overall study management and maintenance of the site file and all data collected from this study. The CI will be responsible for ensuring that all other investigators have received suitable training according to their role in the study.

**Steering group**

The steering group will be regularly throughout the study to review ongoing recruitment, processes, and consider when study amendments might be necessary.

**Other researchers**

All researchers at participating sites will be required to sign the delegation log, as authorised by the PI.

**Patient and Public Involvement (PPI)**

Members of the steering group will meet with the designated PPI group throughout the course of this study. The PPI group will assist with interpretation of data analysis, considering if protocol amendments are necessary, and developing the final study report.

| Protocol Contributors | |
| --- | --- |
| The following people have contributed to the development of this protocol: | |
| **Name:** | **Affiliation and role:** |
| Dr Daisy Wilson  (Chief Investigator)  Dr Thomas Jackson | Clinical Lecturer, University of Birmingham  Honorary Consultant Geriatrician, University Hospitals Birmingham NHS Foundation Trust  Associate Professor of Ageing, University of Birmingham  Honorary Consultant Geriatrician, University Hospitals Birmingham NHS Foundation Trust |
| Dr Carly Welch | Senior Clinical Lecturer, King’s College London  Consultant Geriatrician, Guy’s and St Thomas’ NHS Foundation Trust |
| Dr Helen McGettrick | Associate Professor in Inflammation and Vascular Biology, University of Birmingham |
| Dr Animesh Acharjee | Assistant Professor of Integrative Analytics and Artificial Intelligence and Deputy Program Director of Health Data Science, University of Birmingham |
| Dr Niharika Duggal | Assistant Professor in Ageing and Immunity, University of Birmingham |
| Professor Joao Pedro de Magalhaes | Professor of Molecular Biogerontology, University of Birmingham |
| Professor Simon Jones | Professor in Musculoskeletal Ageing and Deputy Director of the Centre for Musculoskeletal Ageing Research, University of Birmingham |
| Dr Ali Mazaheri | Associate Professor in Neuroscience |
|  |  |
| Dr Jose R Hombrebueno | Assistant Professor in mitochondrial biology and experimental  Ophthalmology, University of Birmingham |
| Professor Thomas Pinkney | Chair of Surgical Trials and Director of the Birmingham Surgical  Trials Consortium, University of Birmingham  Consultant Colorectal Surgeon, University Hospitals Birmingham NHS Foundation Trust |
| Dr Jude Partridge | Consultant Geriatrician, Guy’s and St Thomas’ NHS Foundation Trust  Honorary Senior Clinical Lecturer, King’s College London |
| Professor Claire Steves | Professor of Ageing and Health, King’s College London  Honorary Consultant Geriatrician, Guy’s and St Thomas’ NHS Foundation Trust |
| Professor Janet Lord | Emeritus Professor of Inflammation and Ageing, University of Birmingham |

| Protocol Amendments | | | | |
| --- | --- | --- | --- | --- |
| The following amendments and/or administrative changes have been made to this protocol since the implementation of the first approved version | | | | |
| **Amendment number** | **Protocol version number** | **Date of amendment** | **Type of amendment** | **Summary of amendment** |
| **1** | **3.0** | **11.09.2025** | **Substantial** | **Addition of second EEG measure at visit 3** |
| **2** | **4.0** | **30/10/2025** | **Minor** | **Addition of Be Part of Research Volunteer network to recruitment strategy** |
| **3** | **5.0** | **27/11/2025** | **Substantial** | **Addition of Qualitative Interviews after visit 3**  **Extension of study date**  **Additional of additional collabortaors** |

Study summary

| **Study Title** | REsilience PROmotion with GeRoprotectors: AssessMent of biological effect (REPROGRAM study) |
| --- | --- |
| **Study Registration** |  |
| **Sponsor** | University of Birmingham  0121 414 7618; [researchgovernance@contacts.bham.ac.uk](mailto:researchgovernance@contacts.bham.ac.uk) |
| **Funder** | Wellcome LEAP Dynamic Resilience programme |
| **Study Design** | Open Label, uncontrolled, Non-CTIMP |
| **Study Participants** | Older volunteers – 70 years and older |
| **Planned Recruitment** | 60 participants (20 each agent; 50% female) |
| **Follow Up Duration** | 5 weeks for each participant |
| **Planned Study Period** | June 2024 – December 2026 |
| **Primary Research Question** | Does a three week course of metformin, spermidine, or fisetin reduce the number of senescent cells as measured by SA-β-GAL in adipose biopsies in healthy older volunteers? |
| **Intervention** | Metformin MR – 500mg once a day for one week and then 1500mg once a day for three weeks, oral administration  Fisetin – 100mg once a day for three weeks, oral administration  Spermidine – 15mg once a day for three weeks, oral administration |

Study schema


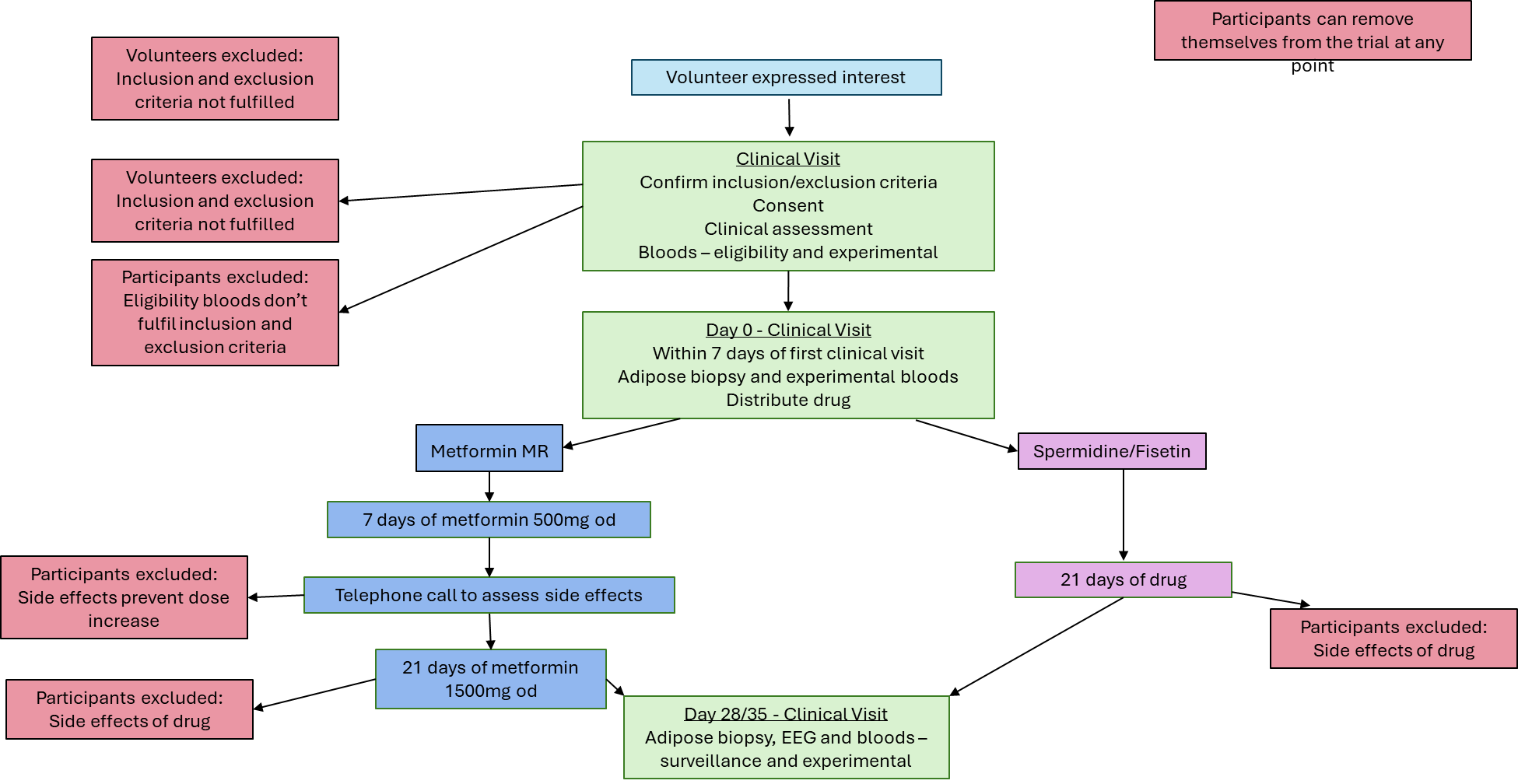


 Day 28/35 Clinical Visit may be + 3 days

Study protocol

**Resilience PROmotion using GeRoprotectors: AssessMent of biological effect (REPROGRAM study)**

1. Background

Doubling of life expectancy in the last 200 years is testament to success of public health and medical advances. However, population ageing presents a major health and social care challenge. As we age, our biological systems and clinical features become increasingly diverse. This diversity can lead to either healthy ageing or development of age-related conditions such as frailty, sarcopenia, and cognitive decline (1). Advancement of age is associated with reduced resilience, hindering the ability to maintain balance and stability when confronted with infections, falls, or surgery (2). This loss of dynamic resilience with age, whilst universally recognised, is poorly defined or understood biologically, limiting our ability to intervene to maintain health in old age.

Advances in ageing biology have enabled the description of key biological mechanisms driving the aged phenotype, the hallmarks of ageing (3), including: reduced mitochondrial fitness, telomere shortening, reduced autophagy, accumulation of senescent cells, microbial dysbiosis and increased systemic inflammation. Importantly, targeting these ageing processes, for example by deleting senescent cells (4), extends healthspan in mice, reducing development of age-related conditions. Geroprotectors are drugs which can affect underlying ageing processes rather than a single symptom or disease. However, there is an absence of robust pharmacodynamic and pharmacokinetic data on their ability to modify the proposed targets in vivo in humans. Additionally, it is unclear which patients should be treated and where in their life-course the drugs will have the optimum effect (5).

This study aims to investigate three different agents, metformin, fisetin and spermidine, and determine their ability to modify biological pathways in vivo in humans. These agents have been chosen as drugs with both evidence of effect on the hallmarks of ageing and good safety profiles in frail, older adults. Metformin, an oral hypoglycaemic agent, has been used safely in frail, older adults for over 60 years (6). Fisetin, a plant flavonol, and spermidine, a natural polyamine, can both be bought as a supplement in the UK and have no reported side effects (7) (8, 9).

Metformin lowers the incidence of multiple age-related diseases, including frailty, as well as all-cause mortality in adults with diabetes (10, 11). In non-diabetic mice and C. elegans metformin extends both lifespan and some indicators of healthspan including muscle strength and gait speed (12) (13). There is evidence in cell lines, drosophila, C. elegans and mice that metformin modulates multiple hallmarks of ageing: nutrient sensing, intracellular communication, proteostasis, genomic instability, mitochondrial function, stem cell exhaustion, epigenetic alterations, telomere attrition and cellular senescence (14). There is some evidence that metformin influences healthspan in non-diabetic adult humans. Laksmi found that metformin improved walk speed in pre-frail adults (15) but the MASTERS trial found no benefit of using metformin as augmentation to resistance training (16). MET-PREVENT (17) and TAME (18) are yet to report on their outcomes of using metformin in older adults with sarcopenia and pre-frailty or frailty and older adults respectively.

Fisetin is found in edible fruits and vegetables such as strawberries, apples and persimmons (19). Fisetin is a senolytic agent and reduces the number of senescent cells in a variety of animal and tissue models (20, 21). Fisetin in mice has been demonstrated to extend both life and healthspan (22). There are several ongoing studies and studys in humans none of which have yet reported (23).

Spermidine is found at high levels in many food products including dry soy beans, chicken liver and blue cheese (24). It is an essential metabolite that exhibits age-dependent decline in a number of models including humans (25). It is believed to effect multiple hallmarks of ageing but the evidence is limited (25). In yeast, C. elgans, mice and rat models spermidine increased lifespan and in rats healthspan (25). Epidemiological studies have demonstrated that diets high in spermidine are associated with lower mortality (26). There are number of ongoing clinical trial investigating spermidine in age related diseases such as hypertension and heart failure (25).

1. Rationale

Patient and public involvement

Patient representatives have been actively involved in the design of this study and will act as members of the steering group throughout the study. Patient representation will be imperative in considering the implications of the study, and will be actively involved in interpretation and dissemination of results.

Rationale for research questions – relevance to NHS patients and healthcare policy

Advancements in medicine have led to increased life expectancy through improved treatment of conditions such as cancer, heart disease, and infections{Loh, 2013 #148}{Statistics, 2017 #149}. However, these improvements in lifespan have not been associated with increases in healthspan (the number of years spent in good health). It is known the outcomes that are important to older people is not always longevity, but rather perceived quality of life. This study aims to determine whether targeted interventions towards underlying biological mechanisms associated with poor resilience can change the biological pathways within a 3 week period. It is undetermined when geroprotectors should be given in an individual’s lifecourse to have the greatest effect but research increasingly supports intervention at an early stage to modify the development of age-related disease and multimorbidity{Lee, 2023 #150}{Kennedy, 2014 #151}. We have previously conducted a study of a multi-component supplement in healthy older adults which demonstrated an improvement in epigenetic age{McGee, 2024 #152}. This is a similar approach to other studys and studies of geroprotector agents: metformin {Walton, 2019 #153} and rapamycin {Chung, 2019 #155}{Kraig, 2018 #156}{Mannick, 2014 #154}

Rationale for study design

This study is a Phase 2a clinical study. It is designed to investigate the effect of the intervention on biological outcomes. The intervention, metformin, spermidine or fisetin, will be administered for three weeks to healthy volunteers. This mimics the time between a diagnosis of localised cancer and curative surgery and will attempt to determine whether these agents could be used effectively in the prehabilitation process to prevent loss of resilience post-surgery. Healthy volunteers are being recruited to the study rather than patients because the biological outcomes of these interventions for this duration are unknown.

1. Research question/aims

Objectives

Primary Research Question

- Does a three week course of metformin, spermidine, or fisetin reduce the number of senescent cells as measured by SA-β-GAL in adipose biopsies in healthy older volunteers?

Secondary Research Questions

- Does a three week course of metformin, spermidine, or fisetin reduce senescence in healthy older volunteers?
- Does a three week course of metformin, spermidine, or fisetin reduce mTOR activation in healthy older volunteers?

Outcomes

Primary Outcome

- Number of senescent cells in adipose tissue measured by SA-β-GAL technique after three weeks of intervention, adjusted for baseline number of senescent cells prior to the intervention.

Secondary Outcomes

- Senescent cell burden in adipose tissue as measured by transcriptomics using SenMayo analysis after three weeks of intervention, adjusted for baseline number of senescent cell burden prior to the intervention.
- Epigenetic age (DNA methylation) after three weeks of intervention, adjusted for baseline epigenetic age prior to the intervention.
- Immunesenescence as measured by IMM-AGE analysis after three weeks of intervention, adjusted for baseline IMM-AGE prior to the intervention.
- Autopahgic flux as measured by flow cytometry after three weeks of intervention, adjusted for autophagic flux prior to the intervention.
- Nutrient sensing as measured by mTOR activation after three weeks of intervention, adjusted for baseline nutrient sensing prior to the intervention.
- Mitochondrial function as measured by Seahorse after three weeks of intervention, adjusted for baseline mitochondrial function prior to the intervention.
- Inflammation as measured by ELISA and multiplex technology after three weeks of intervention, adjusted for baseline inflammation prior to the intervention.
- Microbial composition and functional potential of stool using metagenomics after three weeks of intervention, adjusted for baseline microbial composition and functional potential prior to the intervention.
- Changes in neural oscillatory signatures measured by EEG.

1. Study design and methods of data collection and data analysis

This study is a Phase 2a clinical study. It is designed to investigate the effect of the intervention, metformin, spermidine, or fisetin, on biological outcomes. Additionally, an embedded qualitative interview sub-study will be undertaken with a sub-sample of participants enrolled in the REPROGRAM trial. See section 8 for information about the qualitative sub-study.

Power Calculations

The sample size calculation is based on measurements of SA-β-Gal from a senolytic study in adults (27). The number of participants required at 80% power and a significance level of 0.05 to detect a mean difference of 5% in SA-β-Gal marker is 9. To allow for drop out 10 participants of each sex will be recruited to each intervention (metformin, spermidine, or fisetin), 20 in total. We are recruiting 10 participants of each sex for each intervention as there is some evidence of sex differences with women believed to be more prone to senescence (28).

Data management

Investigator Site Files (ISF) will be maintained for the duration of the study. Source data will be collected on paper CRFs and/or eCRFs on REDCap secure software. Data collected on paper CRFs will be transferred to eCRFs on REDCap. Paper CRFs and consent forms will be held securely at the University Hospitals Birmingham NHS Foundation Trust and will not leave this site. These should be filed within the ISF, or the location of these should be stated clearly if filed elsewhere. Electronic ISFs may be used, but hard copy case report forms and completed paper CRFs must be securely maintained on site. Hospital sites will be responsible for the secure archiving of data. Data will be stored as per the Data Protection Act, 2018.

Data will be securely uploaded through REDCap, which is secure encrypted data management software. Access to REDCap will be provided to users at an appropriate level, with roles setup accordingly. Patient identifiers including date of birth, and participant names will be securely recorded within REDCap for individual participants. Postcodes will be recorded to enable estimation of multiple deprivation indices. Data will be archived for ten years.

Data analysis

Data analysis will be conducted using appropriate statistical software packages by individuals with appropriate expertise. It is envisaged that simple statistical methods (such as t-tests and Mann-Whitney dependent on the normality of the data) will be complemented with more complex analysis using bio-informatic techniques.

Data Protection

All laboratory specimens and CRFs will be identified in a manner designed to maintain participant confidentiality. Laboratory specimens will arrive at the lab in a pseudo-anonymised form. The anonymisation will be done by the person taking the sample. All EEG data will be securely stored in line with institutional data protection policies. EEG recordings do not contain identifiable information and cannot be used to recognise or identify individual participants. The data reflects brainwave patterns only and is stored securely without any personal details attached. All records will be kept in a secure storage area in the Inflammation Research Facility with limited access to study staff only. Information will not be released without the written permission of the participant, except as necessary for monitoring and auditing by the Sponsor, its designee or Regulatory Authorities. The CI and study staff involved with this study will not disclose or use for any purpose other than performance of the study, any data, record, or other unpublished, confidential information disclosed to those individuals for the purpose of the study.

The CI and study staff involved with this study will comply with the requirements of the Data Protection Act 2018 with regard to the collection, storage, processing and disclosure of personal information and will uphold the Act’s core principles. Access to collated participant data will be restricted to the CI and appropriate study staff.

Computers used to collate the data will have limited access measures via user names and passwords.

Published results will not contain any personal data that could allow identification of individual participants.

GP Notification

We will inform the GP of the participants involvement in the study and any clinical information that would be beneficial for the participants’ ongoing healthcare.

Intervention

Participants will be randomly allocated to either Metformin MR, spermidine or fisetin on Day 0 Clinic Vist. Participants will be randomised by the study team using software which will perform stratified randomisation. There will be an equal number of men and women within each drug group and 20 participants within each group.

**Table 1 – Intervention, administration, dose and supplier**

| **Intervention** | **Administrative route** | **Dose** | **Supplier** |
| --- | --- | --- | --- |
| Metformin MR | Oral | - 500mg once a day for 7 days. - Telephone call for assessment of side effects. If no concerns proceed to - 1500mg once a day for 21 days | Clinical Research Facility Pharmacy |
| Spermidine | Oral | - 15mg for 21 days | Longevity Box |
| Fisetin | Oral | - 100mg for 21 days | Longevity Box |


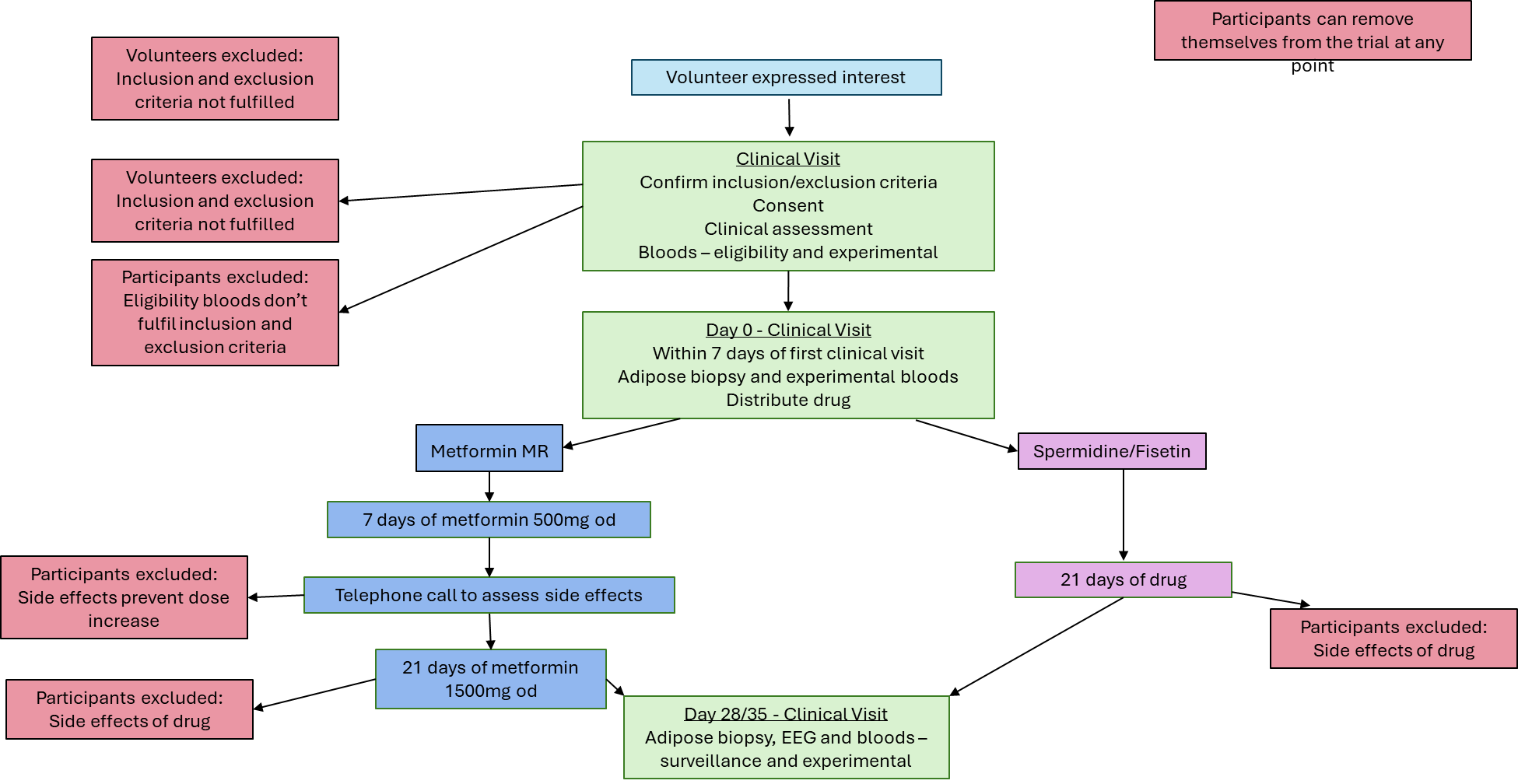


1. Study setting

This is single site study at the University Hospitals Birmingham NHS Foundation Trust. Healthy volunteers will be recruited using established networks. The Clinical Visits will be in the Clinical Research Facility (CRF) at the University of Birmingham. This is based at Queen Elizabeth Hospital Birmingham.

1. Participant Recruitment

Eligibility Criteria

The study population is healthy older people.

Inclusion criteria

- Age ≥70 years.
- Ability to provide informed consent.
- Able to travel to the clinic for initial and subsequent evaluations.
- Ability to understand spoken and written English

Exclusion criteria

- Current smokers, or ex smokers who have stopped within the last 12 months, or are using nicotine replacement products.
- History of diabetes (type 1 or 2), or untreated vitamin B12 deficiency.
- Untreated thyroid disorder.
- Active cancer - currently on treatment or palliative .
- Major illness, surgery or trauma in last 60 days.
- Recent infection in last 60 days.
- Allergies or intolerance to any of the drugs to be studied.
- Any contraindication to metformin MR, as listed in the current summary of medicinal product characteristics for metformin MR.
- Any medication which significantly interacts with metformin MR, as listed in the current summary of medicinal product characteristics for metformin MR.
- Currently taking any of the following medications, which confound the effects of inflammation: Tamoxifen, Cyclosporine A, immunosuppressants or Anti TNF inhibitors, Non-Steroidal Anti-Inflammatory Drugs (NSAIDs).
- Currently taking any of the following anticoagulant drugs (blood thinners): Warfarin, Direct oral anticoagulant drugs (DOACs), low molecular-weight heparin, clopidogrel, or high-dose aspirin. Patients taking low dose ≤75mg aspirin only may be recruited.
- Individuals at risk of bleeding complications, including those with inherited bleeding disorders (such as haemophilia), or previously unexplained haemorrhage.
- Unable or unwilling to maintain current lifestyle throughout study such as eating habits, exercise habits, etc.
- Estimated glomerular filtration rate <45 mL/min/1.73 m2 by the Modified Diet in Renal Disease 4 (MDRD4) or Chronic Kidney Disease - Epidemiology collaboration (CKD-EPI) equation.
- History of diarrhoeal illness within the last three months (>48 hours of Bristol stool chart grade 6 or 7).
- Liver function tests (bilirubin, alanine aminotransferase or alkaline phosphatase) >3× upper limit of normal.
- Symptomatic chronic heart failure, diagnosed according to European Society of Cardiology guidelines (asymptomatic left ventricular systolic dysfunction will not be an exclusion criterion).
- Life expectancy of <3 months as adjudicated by the local Investigator.
- Previous inclusion in study investigating effects of ‘geroprotector’ medication in last six months.
- Allergy to local anaesthetic.
- Coeliac disease (spermidine only) {Loh, 2013 #148}

Recruitment target

The target recruitment is 60 healthy volunteers to start the medication. It is anticipated that following inclusion bloods a few volunteers may have to be excluded as they will no longer fulfil the inclusion and exclusion criteria.

Recruitment technique

Participants will be invited by contact through the 1000 Elders database. The 1000 Elders is a database of research active healthy older people who have consented to be contacted about studies run through the University of Birmingham. Names, addresses and contact details (telephone and email) are stored complying fully with the Data Protection Act 2018 legislation. The invitation will be in the form of an email. This will include an attached PIS, summary of the study, and an invitation to contact the research team if they would be interested in taking part in the study. The study will also be advertised through a public facing website and through local networks.

The Be Part of Research Volunteer network will send out study information and study contact details on our behalf to their network of volunteers. Interested volunteers will contact the study team for further information.

Participants will be reimbursed £200 for their involvement within the study. Payment will be made to all those recruited to the study. Anyone who attends for the initial visit but is not eligible for recruitment to the study or changes their mind after discussion with the study team will receive reimbursement for their travel to the visit – maximum of £20.

Recruitment

Potential participants will contact the study team via email or telephone to express an interest to join the study. Potential participants will then be invited to the Clinical Research Facility at Queen Elizabeth Hospital Birmingham for the initial visit.

Consent

At the initial visit eligibility will be confirmed and informed consent will be gained for all activities in the study. The nature and objectives of the study will be explained, and potential risks discussed. This information will be presented in a written patient information sheet (PIS) sent to potential participants prior to the in person visit, giving participants time to consider their participation. The process for withdrawal, including the subsequent impact on data and sample storage will be described.

At this point capacity to consent to be in the study will be assessed using the Mental Capacity Act. Participants will then sign a written consent form, and a copy of the information sheet and consent form given to them, and other copies stored in the study file. At the subsequent visit the participant’s willingness to continue in the study will be ascertained. Throughout the study the participant will have the opportunity to ask questions about the study. Any new information that may be relevant to the participant’s continued participation will be provided.

1. Study procedures

Table 1 demonstrates the timing of the individual assessments/procedures that will be conducted as part of this study.

Table 2 – Timing of individual procedures involved in this study

|  | **Initial assessment** | **Day 0 Visit** | **Day 7 - Phone Call (Metformin MR only)** | **Phone Call**  **(Day 10/17)** | **Post-intervention assessment**  **(Day 28/35)** | **Qualitative Interviews** |
| --- | --- | --- | --- | --- | --- | --- |
| Timing |  | Up to 7 days post initial assessment |  | +/- 3 days of 10/17 | +3 days of 28/35 | Up to 3 months from Day 28/35 |
| Informed consent | X |  |  |  |  | x |
| Co-morbidities and medications | X |  |  |  |  |  |
| Barthel Index | X |  |  |  |  |  |
| Nottingham Extended Activities of Daily Living | X |  |  |  |  |  |
| Frailty assessment | X |  |  |  |  |  |
| Handgrip strength | X |  |  |  |  |  |
| Short Physical Performance Battery | X |  |  |  |  |  |
| Three-day food record | X |  |  |  |  |  |
| Ultrasound quadriceps | X |  |  |  |  |  |
| Bioelectrical Impedance Analysis | X |  |  |  |  |  |
| Montreal Cognitive Assessment (MoCA) | X |  |  |  |  |  |
| Stool collection | X |  |  |  | X |  |
| EEG | X |  |  |  | X |  |
| Venepuncture | X | X |  |  | X |  |
| Adipose biopsies |  | X |  |  | X |  |
| Adverse event reporting | X | X | X | X | X |  |
| Drug distribution |  | X |  |  |  |  |
| Compliancy pill count |  |  |  |  | X |  |
| Qualitative Interview |  |  |  |  |  | X |

Co-morbidities and medications

Co-morbidities and medications will be recorded using check boxes for frequently encountered conditions and medications with the option for free text to record less common co-morbidities and medications.

Handgrip strength

Handgrip strength will be measured using a handheld dynamometer (Jamar, Takei Grip D, or other calibrated device). Where the participant can sit out in a chair, handgrip strength will be measured with the arm flexed at 90^O^ at the elbow and the forearm supinated. If the participant cannot sit out and measurements are taken in the bed, this will be recorded and measurements will instead be performed in the most upright position that is feasible. Participants will be asked to “squeeze as hard as [they] can”. Handgrip strength will be measured twice on each side and the best recording of all will be used for analysis (29).

Short physical performance battery

The Short Physical Performance Battery (SPPB) is a standardised measure of physical performance that has been shown to be sensitive to change and provides an objective measure of physical function (30). SPPB consists of usual gait speed, side-by-side stand, semi-tandem stand, tandem stand, and five chair stands. A total score of 12 is derived, with a lower score representing reduced physical performance. SPPB will be measured within 48 hours of admission and 48 hours of discharge. Gait speed will be measured by asking the participants to walk a four-metre course at their “usual pace”. Participants will be asked to complete five chair stands “as quickly as [they] can” (see Appendix). If it is not possible to measure gait speed or any part of the SPPB at any planned visit the reasons for this will be recorded (e.g., if either the investigator or the participant considers it unsafe to do so). The need or use of a mobility aid will be recorded. It is recommended that the SPPB should be performed after assessment of muscle quantity/quality (ultrasound quadriceps and bioelectrical impedance analysis), to ensure that these assessments are not affected the effect of exercise.

Activities of Daily Living (ADL) assessment

The Barthel Index (Appendix) (31) and Nottingham Extended Activities of Daily Living (see Appendix) (32) will be completed for participants. This information can be gathered from asking the participant themselves. The Nottingham Extended Activities of Daily Living can be provided to the participant to complete themselves as a questionnaire where they are able to do so.

Frailty assessments

Frailty will be defined using three definitions: 1) Clinical Frailty Scale 2) Fried phenotype 3) Frailty Index. The Clinical Frailty Scale (CFS) assesses frailty on a 9-point scale (Appendix). CFS will be assessed by the investigator by reviewing the clinical records and an overall holistic assessment of the participant, considering the results of the ADL assessment above. The Fried phenotype will be assessed utilising information collected from assessment of handgrip strength and gait speed, and directly asking the participant about weight loss, physical activity, and self-reported exhaustion (Appendix). The frailty index will be calculated from information collected in the study elsewhere, with direct questioning where necessary (Appendix).

Cognitive testing

Cognitive testing will be performed using the Montreal Cognitive Assessment (MoCA) (33).

**Electroencephalography**

Electroencephalography (EEG) recordings will be conducted using a saline-soaked 32-channel EEG net, specifically designed for a quick and gel-free application. The EEG device is a CE marked and approved by University Hospitals Birmingham (UHB). The EEG measurements will be taken during the performance of cognitive paradigms, as well as during resting state. These recordings will provide valuable insights into brain activity patterns. The cognitive tasks performed during the EEG recordings will include the Attention Network Task, which has proven to be effective in studying attentional processes, and a versatile language comprehension paradigm. These tasks demonstrate sensitivity to age-related and early cognitive decline-associated changes in neural oscillations so are appropriate for maximizing sensitivity to intervention-related changes in older adults. The data from the EEG will be used to support mechanistic research into intervention related changes in neural oscillations in older adults. It will not be used to evaluate the safety or performance of the EEG device and it will not be used for diagnosis, monitoring or treatment guidance.

Stool sample collection

Stool samples will be collected in sterile containers. Participants will be provided with a sterile container and protective delivery bag to either post the sample directly to the research team or hand deliver to the hospital at the earliest convenience. Stool samples will be frozen at -80^o^C prior to analysis. Stool samples will be transferred for storage at the Department of Twins Research, King’s College London (our partners in a wider Wellcome Leap grant).

Venepuncture

Blood will be collected peripherally using BD vacutainers. A maximum of 70mL of blood will be taken at any one time. Blood samples will be used for eligibility, surveillance and further analysis. Eligibility and surveillance blood samples will be analysed for: full blood count, liver and renal function, vitamin B12 and metabolic acidosis. Surveillance bloods to be completed as per NICE guidelines. Samples will be prepared to enable further analysis and frozen at -80^o^C (this will include whole blood, peripheral blood mononuclear cells, serum, and plasma samples).

Adipose biopsies

Adipose tissue will be obtained from the abdomen. An aseptic technique will be used. Local anaesthetic will be infiltrated to the skin prior to biopsy. Biopsies should not be performed in participants taking anticoagulants or clopidogrel, but may be performed in participants taking only low dose aspirin 75mg.

Samples will be prepared to enable further analysis and frozen at -80^o^C and stored at the University of Birmingham.

Ultrasound quadriceps

Ultrasound measurement of quadriceps muscle will be performed using two-dimensional B-mode ultrasonography with a linear probe. Participants will be positioned in a semi-upright position with their knees resting at 10-20° using a firm wedge. Participants will be asked which leg they consider to be dominant and this will be recorded. Participants will be advised to relax their muscles. The distance from the greater trochanter to the lateral joint line of the knee will be recorded and a mark will be placed on the skin mid-way between the two points on both sides. All ultrasound measurements will be taken in line horizontally with these marks (37).

Muscle thickness measurements will be taken with the probe held in transverse position, perpendicular to the lateral border of the thigh (37). Contact gel will be applied to the skin. The depth will be adjusted until the femur and overlying structures are visible. The Rectus Femoris (RF) will be identified by locating its borders. The probe will be positioned such that the widest area of the RF appears over the midpoint of the femur. A frozen image at this location will be taken with the probe held in maximal relaxation. Thickness measurements of the subcutaneous tissues (SC), RF and Vastus Intermedius (VI) taken in a vertical line will be recorded, not including the fascia. Three frozen images will be used for all patients and a further image will be taken if there is greater than 10% variability between measurements. Frozen images taken which the research team deem to inappropriate due to showing unclear borders or grossly non-perpendicular imaging may be discounted from inclusion.

The mean of each reading will be used for analysis. BATT will be calculated as the total thickness of right VI + right RF + left VI + left RF. BATT: SC ratio (BATT-SCR) will be calculated as BATT divided by the total thickness of right SC + left SC (38). Where possible, the cross-sectional area of the right and left RF will be measured in the transverse plane (39). Each of these measurements will be repeated at both clinic visit with participants. All measurements will be performed by an investigator with training in taking these measurements. The intra-rater and inter-rater reliability of BATT has been previously shown to be excellent when using the same protocol (38). BATT will be used alongside walk speed and grip strength to diagnose sarcopenia (Appendix).

A further image will be taken with the probe held in the longitudinal position at each visit. Images will be saved and downloaded for assessment. Echogenicity will be determined using grey-scale analysis on Image J software (37, 38).

Bioelectrical Impedance Analysis

Bioelectrical impedance analysis (BIA) will be measured at each visit using the Bodystat Quadscan 4000. This procedure will not be performed if the participant has an implanted permanent pacemaker or defibrillator. The investigator will determine if a cardiac device is present by asking the participant and/or consultee where possible, reviewing medical notes, including imaging where appropriate, and externally examining the participant. Participants with cardiac devices may still be included in the study, but this procedure will not be performed. Two electrodes will be placed on the right foot; one below the base of the toes and the other on the ankle between the medial and lateral malleoli. Electrodes will be placed sideways so that the non-stick electrode connector is facing the researcher. The red alligator clip will be attached to the electrode nearest the toes and the black to the one at the ankle. A further two electrodes will be placed on the right hand; one behind the knuckles and the other on the wrist next to the ulnar head. The red alligator clip will be attached to the electrode nearest the fingers and the black to the one at the wrist. Height and weight measurements should be obtained (these can be extracted from clinical records if available or measured by the research team). During hospitalisation, weight may be estimated if it is not possible to obtain new readings, but this should be documented by the research team. Key participant information (height, weight, age, sex) will be entered onto the device and results will appear on screen. All returned results will be recorded. This will include fat weight, lean weight, total body weight, dry lean weight, skeletal muscle mass (SMM), and extracellular water (ECW) and intracellular water (ICW).

Three-day food record

Participants will be supplied with a three-day food record at their research appointments and asked to complete this for the next three days. They will then post this back to the research team. This will be used to estimate daily calorific and protein intake at each assessment, adjusted for the participant’s weight at that timepoint.

Adverse Event Reporting

Participants will be asked about any adverse events that occurred during the study period. These will be recorded and reported appropriately. During the telephone call mid-way through the study intervention period participants may be excluded from continuing in the study.

On day 7 all participants taking metformin will be rung and asked about side effects to the drug and to determine whether to withdraw the participant at this point or to increase the dose to the study dose of 1500mg for 21 days.

Drug Distribution

Participants will be supplied with the drugs they are taking for the study and counselled on possible side effects and the appropriate course of action. They will be supplied with all the drugs at the Day 0 Clinical Visit by a medical professional. The metformin will be prescribed by a study doctor and supplied by hospital pharmacy on the 2^nd^ research clinic visit. Spermidine and fisetin are nutraceuticals and therefore do not need a prescription. They will be supplied with instructions on how to take them.

Compliancy Pill Check

Participants will be asked to bring back the boxes and packets of the drugs they have been supplied with to check their adherence to the drugs during the study period. A member of the study team will check the boxes to see whether all the tablets have been taken. They will record the number of tablets left. They will also ask the participant if they took all their tablets as directed and if not why. The participants will also be asked to complete the drug record.

1. **REsilience and Frailty Research – Lived Experience of Community-dwelling Older AdulTs (REFLECT Study)**

8.1 Background

Older adults, particularly those living with frailty and from minoritised ethnic communities, remain under-represented in clinical research, limiting both the generalisability and equity of trial findings (52). Recruitment to REPROGRAM has been less diverse than anticipated, drawing mainly on the Birmingham 1000 Elders cohort and, more recently, on the NIHR *Be Part of Research* volunteer registry (53). This pattern mirrors wider challenges in enrolling older adults living with frailty into research and raises questions about how potential participants understand, experience and judge invitations to take part in frailty and geroprotector trials. Understanding these experiences is important for improving the inclusivity, acceptability and feasibility of future trials, consistent with NIHR INCLUDE guidance on under-served groups and MRC/NIHR guidance on complex interventions (52,59).

REFLECT is an optional qualitative interview sub-study nested within REPROGRAM. It focuses on the lived experience of community-dwelling older adults who have taken part in the trial.

8.2 Aims

The qualitative sub-study aims to:

1. Identify the practical, social, cultural and emotional factors – mapped to capability, opportunity and motivation – that make participation in frailty research easier or harder, including what stops people from taking part.
2. Identify the capability, opportunity and motivation factors that made participation in REPROGRAM easier or harder, including what nearly stopped people from taking part.
3. Generate participant-informed recommendations to improve invitation methods (including the use of NIHR *Be Part of Research* and Birmingham 1000 Elders), study information and trial procedures in future geroprotector and frailty trials.

8.3 Design and setting

REFLECT is a single-centre, qualitative interview study nested within REPROGRAM. Semi-structured interviews will be conducted with approximately 15–20 REPROGRAM participants. Purposive sampling will seek diversity in sex, age, intervention group, baseline frailty, ethnicity, recruitment route and socioeconomic circumstances, where feasible.

Interviews will follow best-practice guidance for qualitative interviewing in health and medical education, including careful preparation, piloting and iterative refinement of the topic guide with a PPI group, explicit attention to rapport and power dynamics, and planning for the management of emotional distress (54). Interviews will take place after the Day 28/35 post-intervention visit, in person, and within three months of V2, in the Inflammation Research Facility.

8.4 Participant identification, recruitment and consent

Eligibility for REFLECT is restricted to participants already enrolled in REPROGRAM who have completed the Day 28/35 post-intervention visit and retain capacity to consent. At the time of consent for the main REPROGRAM study, participants will be asked whether they are willing to be contacted about future optional sub-studies. This preference (yes/no) will be recorded on the updated main study informed consent form. Only participants who have agreed to further contact will be approached about REFLECT.

From this pool, the research team will use purposive sampling to achieve a maximum-variation sample. Eligible participants will be approached by the clinical research team by email, telephone or during the Day 28/35 visit and provided with a separate qualitative Participant Information Sheet describing the sub-study. They will normally be given at least 24 hours to consider participation before arrangements are confirmed. An additional £30 would be offered to REFLECT participants for their time.

Written informed consent for the qualitative interview will be obtained separately from consent for the main trial. Participants will be reminded that participation is voluntary, that they may decline to answer any question or stop the interview at any time, and that withdrawal from REFLECT will not affect their participation in REPROGRAM or their clinical care.

8.5 Data collection

Interviews will last approximately 45–60 minutes. Topic-guide domains will include:

- initial reactions to being approached about REPROGRAM (including recruitment route);
- understanding of the trial and geroprotector interventions;
- perceived risks, burdens and benefits;
- practical, social and emotional barriers and enablers to participation; and
- participants’ suggestions for improving information materials, recruitment strategies (including digital registries such as *Be Part of Research*) and study procedures (e.g. adipose biopsy, stool sampling, EEG).

Interviews will be audio-recorded using encrypted devices and transcribed verbatim. The interviewer will keep brief field notes on context, salient non-verbal cues and reflexive observations. If participants become distressed, the interview will be paused or stopped as appropriate. Participants will be offered time to recover, the option not to continue, and signposting to appropriate support if required. Any substantial distress will be documented and reported in line with Good Clinical Practice and sponsor requirements.

8.6 Sample size and information power

Rather than aiming for a fixed notion of “data saturation”, final sample size will be guided by the concept of information power (57). Approximately 12–20 interviews are expected to provide adequate information power given:

- focused study aims;
- a relatively specific clinical population (older adults enrolled in REPROGRAM);
- the use of a coherent theoretical framework (COM-B) (56); and
- an in-depth, iterative analytic strategy.

The final number will balance the richness and diversity of participants’ accounts with pragmatic considerations.

8.7 Data management and protection

Audio files will be transferred as soon as practicable after data collection to encrypted, password-protected University of Birmingham servers and deleted once checked transcripts are available. Transcripts will be pseudo-anonymised before analysis; names and other potentially identifying details will be removed or generalised. Documents linking participant identities to study identification numbers will be stored separately from qualitative data and accessible only to authorised members of the research team.

Qualitative data (transcripts, coding files and analytic memos) will be stored on secure institutional drives and/or within approved qualitative analysis software (e.g. NVivo), with access restricted to named study team members. Data will be archived for ten years in line with the wider REPROGRAM study and managed in accordance with Good Clinical Practice, the Data Protection Act 2018, UK GDPR and local NHS/University information-governance policies.

8.8 Data analysis

Interview transcripts will be analysed thematically using a reflexive thematic analysis approach (58). Analysis will proceed through iterative stages of:

- familiarisation with the data;
- generation of inductive codes capturing salient features of participants’ accounts;
- organisation of codes into candidate themes and sub-themes;
- review and refinement of themes against coded data and the full dataset; and
- production of an analytic narrative that addresses the study aims.

The COM-B model (Capability, Opportunity, Motivation–Behaviour) will be used as a sensitising framework to explore how cognitive, physical, social and emotional factors shape decisions about research participation (56). A case-by-theme matrix will be developed to support systematic comparison within and across participants, drawing on principles from the Framework Method (55). To enhance reflexivity and credibility, a subset of transcripts will be independently read and coded by more than one member of the research team, with regular analytic discussions to review interpretations, consider alternative explanations and reflect on researcher positionality. Qualitative data management and coding will be supported by NVivo.

8.9 Outputs, dissemination and integration with the main trial

Findings from REFLECT will inform at least one peer-reviewed manuscript describing older adults’ motivations for participation, perceptions of study procedures (for example adipose biopsy, stool sampling and EEG), tolerability of the interventions, and views on the future use of geroprotectors in clinical care. Qualitative insights will also be integrated, where appropriate, into mixed-methods outputs from REPROGRAM to contextualise and interpret the biological and mechanistic findings and to guide optimisation of recruitment strategies, information materials and trial procedures in future geroprotector and frailty trials, in line with NIHR INCLUDE and MRC/NIHR guidance (52,59). Key qualitative themes will be summarised in accessible language within participant result summaries and public-engagement activities, with input from the PPI group to ensure clarity and relevance.

1. Detailed laboratory methodology for mechanistic studies

Laboratory based assays will be conducted at University of Birmingham, stool microbiome and metagenomics which will be conducted at the Department of Twins Research King’s College London. We may send samples to our research partners for analysis that cannot be completed at University of Birmingham or Kings College London. They will not have access to personal data. Analysis of the data will be conducted at the University of Birmingham.

Stool microbiome and metagenomics

Dysbiosis is a hallmark of ageing that influences health (41). Microbial composition and functional potential will be assessed through metagenomics in stool to identify biomarkers of resilience using state of the art metagenomic sequencing (Illumina NovaSeq6000 DX sequencer). This will include bacterial taxonomic and functional profiling, and virus profiling (42). This will allow assessment of the changes of the gut microbiome following the intervention with either metformin, spermidine or fisetin. This is considered particularly important given the known effect of metformin on gastrointestinal tract with diarrhoea being a commonly reported side effect ((Relative risk=2.445 95%CI [1.656, 3.609], p=0.0001) compared to controls.)

DNA methylation, immune system ageing, compromised autophagy, mitochondrial function, nutrient sensing and stem cell exhaustion

Epigenetic age and epigenetic response (at days 0, 30 and 90) will be assessed from isolated whole blood DNA using the Illumina EPIC array and published algorithms **(43)**. Immunesenescence will be assessed by deriving the IMM-AGE score **(36)** by immunostaining and flow cytometry analysis of isolated PBMCs identifying 8 immune cell types (total T cells, naive CD4 T cells, effector memory CD4 and CD8 T cells, EMRA CD8 T cells, CD28− CD8 T cells, CD57+ CD8 T cells and regulatory T cells). Epigenetic age and immunesenescence are associated with risk of morbidity and mortality. Immunesenescence has been shown in mice to be a key driver of frailty **(44)** and in humans to predict impaired resilience in patients following traumatic injury **(45)**. Autophagic flux assessed by LC3-II breakdown by flow cytometry will determine compromised autophagy from whole blood **(46)**. T cells will be assessed for mTOR activation (by phosphoflow cytometry) and mitochondrial function (measuring cellular energetics using a Seahorse system) as further hallmarks of ageing. Stem cell exhaustion, a hallmark of ageing, will be assessed in mesenchymal stem cells (MSCs) isolated from peripheral blood, to evaluate multipotency, senescence and metabolic plasticity.

Adipose tissue to assess cellular senescence and tissue-based resilience biomarkers

Transcriptomics analysis (bulk RNAseq), will allow assessment of senescent cell burden using the SenMayo analysis (47). This will be used to identify associations between dynamic resilience and canonical signalling pathways that mediate muscle mass and myogenesis (Ingenuity Pathway Analysis) and adipogenesis linked with dynamic resilience or progression of frailty. Protein coding and non-coding RNAs associated with resilience will also be assessed. Primary adipose-derived stem cells (ADSC will be isolated from a third of the tissue and assessed immediately for autophagic flux and mitochondrial function or frozen for later use. The remaining snap frozen tissue will be analysed histologically to confirm the presence of senescent cells (Lamin B1, SA-β-Gal, TAF) and lipid accumulation (e.g., Oil Red O staining).

Inflammatory and senescence markers

Analysis of frozen serum using ELISA and multiplex technology will allow assessment of systemic inflammatory and senescence markers.

1. Safety reporting

An adverse event (AE) is any untoward medical event affecting a clinical study participant. Each initial AE will be considered for severity, causality or expectedness and may be reclassified as a serious event or reaction based on prevailing circumstances.

An adverse reaction (AR) is where it is suspected that an AE has been caused by a reaction to the study supplement

A serious adverse event (SAE), serious adverse reaction (SAR) is any AE, AR or UAR (unexplained adverse reaction) that at any dose:

• results in death

• is life threatening

• requires hospitalisation or prolongation of existing hospitalisation

• results in persistent or significant disability or incapacity

• is a congenital anomaly or birth defect

• Or is otherwise considered serious

Note: Hospitalisations for treatment planned prior to consent and hospitalisation for elective treatment of a pre-existing condition will not be considered as an SAE. However, any adverse events occurring during such hospitalisation will be recorded. Adverse events, adverse reactions and serious adverse events will be reported by participants or other health care professionals.

All AEs and SAEs will be recorded from the time a participant consents to join the study until the last sstudy visit. Participants with unresolved AEs at the last study visit will be followed up until resolution or 30 days after last visit of that participant, whichever is sooner.

The CI, or delegate will ask about the occurrence of AEs and hospitalisations at the second study visit (day 28/35). AEs will be recorded on the AE Log in the CRF. SAEs will be submitted on an SAE form to the Chief Investigator and study team within 24 hours of becoming aware of the SAE. SAEs will be assessed for expectedness and causality by the Investigator. The Sponsor will be informed of any reportable SAEs. The evaluation of expectedness will be made based on the knowledge of the reaction and the relevant product information.

1. Ethical and regulatory considerations

Assessment and management of risk

Physical risks

*Burden of time*

Burden of time is subjective and will vary between participants. Participants will be given time to complete the assessments and to ensure they do not feel rushed, whilst also reassuring them that they can stop or withdraw at any time.

*Burden of completing tasks*

Some participants might find completion of the SPPB and other assessments particularly burdensome, due to fatigue. Participants will be given sufficient time and opportunity to complete these tasks. Where participants were unable to complete tasks, this will be recorded along with the reason for being unable to do so.

#### Risk of falls

Falls are common in older adults, and although steps can be taken to prevent falls, this is not always possible. When performing tests of mobility, the investigator will be responsible for first assessing that the environment is safe (e.g., the floor is not slippery, no obstacles present, the participant is wearing appropriate footwear and sensory aids if necessary, walking aids are provided if needed). If either the investigator or the participant do not feel safe to perform any tests of physical performance then they will not proceed to do so, and this will be documented on the CRF. If the participant does fall, then the investigator will abide by the Queen Elizabeth Hospital Birmingham site policy for manual handling and care of the falling patient. A clinical assessment will then be performed by the investigator to assess for injury and cause for fall. This will be documented in their medical notes, communicated to the usual care team, and an incident form sent as per trust policy.

#### Venepuncture

Participants may find venepuncture uncomfortable, and this may leave a bruise afterwards. A maximum of 70mL of blood will be taken at any time. Some participants may feel faint following having venepuncture performed. Vasovagal syncope (feeling faint whilst having blood taken) is by definition self-limiting so will resolve spontaneously. We will minimise the risk of vasovagal syncope occurring by ensuring the participant’s have blood taken in the specially designed phlebotomy chair at the Institute of Inflammation and Ageing which keeps the feet elevated during phlebotomy. It is also possible to lie the participant flat in the phlebotomy chair so should a vasovagal syncope episode occur it can be resolved quickly by restoring blood flow to the brain.

#### Adipose biopsies

The risks of these procedure are pain, infection, bleeding and local nerve damage (rare). Pain following the procedure will be minimised by using local anaesthetic and advising participants to take simple analgesia such as paracetamol afterwards. Aseptic technique using a sterile field and gloves will be used to minimise the risk of infection. The procedure will not be performed on participants who are taking anticoagulants or clopidogrel, or who have a known bleeding disorder.

*Medical Emergencies*

The Clinical Visits will all occur at the Clinical Research Facility at the University of Birmingham which is located within the Queen Elizabeth Hospital Birmingham. Staff are trained in the recognition and management of medical emergencies and there are procedures in place to manage medical emergencies which may take place during any study or study visit. If required the management of an unwell participant can be escalated to include the NHS medical crash team and a participant can be transferred to an NHS location in the Queen Elizabeth Hospital Birmingham for further assessment and management.

*Electroencephalography*

The risks associated with the saline-based EEG are minimal. Participants may find the task-based EEG slightly tedious due to its repetitive nature, but no significant discomfort is expected.

Diagnosis of previously unknown conditions

Conditions that were previously unknown to the participant may be identified during the process of research data collection; this could be considered both a beneficial and unfavourable consequence. In the case of potentially significant findings, these will be explained to the participant, including the potential consequences and further investigations or treatment that might be indicated. These findings will be relayed to the participants’ General Practitioners (GP) or secondary care medical team, who will be advised to arrange further management or to refer to appropriate specialties; this is usual practice in the United Kingdom (UK). Specific consent for findings to be communicated with their GP or medical team will be gained from the participant; the GP will be informed routinely of recruitment to this study. As the researchers will have a dual role as clinicians working within the NHS trust, it may sometimes be appropriate for the clinicians to arrange further investigations, management, or follow-up, but such decisions should be clearly communicated to the wider NHS team. It should be clarified to the participant that any investigations or management are separate from the research study and are part of NHS care.

The study will fit within existing ethical and regulatory frameworks. Participants are healthy volunteers, and not recruited as NHS patients. However, as the recruitment and assessments will take place within an NHS institution we will secure approval through the Health Research Authority (HRA) and an NHS Research Ethics Committee (REC).

Drug Intervention

Metformin has a long history of use in older adults and the safety profile is good (6). Metformin will be used off-licence. It is licensed only for use in type 2 diabetes but is also commonly used for polycystic ovary syndrome and type 1 diabetes. The drug will be started slowly and the target dose reached over a week to minimise side effects. There will be a telephone call after a week to assess any side effects and to decide whether to continue with the intervention or to withdraw the participant from the study. Spermidine and fisetin are supplements which are available “over the counter” in the UK. The doses have been chosen to mimic doses in recent studies (ref – Senokowitsch, 2023) and also available supplements. There are no known side effects reported in the literature (Wu, 2020, spermidine – a comprehensive review of spermidine)(9).

**Table 3 - Assessment and management of risk**

| **Intervention** | **Risks** | **Amelioration of Risk** |
| --- | --- | --- |
| Metformin MR | **Side effects as listed in British National Formulary (BNF)**  **Common or very common**  Abdominal pain; appetite decreased; diarrhoea; gastrointestinal disorder; nausea; taste altered; vitamin B12 deficiency; vomiting  **Rare or very rare**  Hepatitis; lactic acidosis (discontinue); skin reactions | Metformin MR will be started at a low dose of 500mg once a day as advised by BNF. This will reduce the risk of gastrointestinal side effects.  Participants with a diagnosis of symptomatic heart failure will be excluded to reduce the risk of lactic acidosis.  Vitamin B12 levels will be checked prior to commencing the drug intervention and following completion of the study to diagnose deficiency. Participants with a deficiency prior to starting will be excluded from participation.  Surveillance blood tests will be checked one week after discontinuing metformin MR to ensure there has been no impact on liver function. |
| Spermidine | No known side effects | Surveillance blood tests will be checked one week after discontinuing spermidine to ensure there has been no impact on organ function |
| Fisetin | No known side effects | Surveillance blood tests will be checked one week after discontinuing fisetin to ensure there has been no impact on organ function. |

Research Ethics Committee (REC) and other Regulatory review & reports

Before the start of the study, a favourable opinion will be sought from a REC (Research Ethics Committee) and the Health Research Authority (HRA) through the National Research Ethics Service for the study protocol, informed consent forms and other relevant documents. All correspondence with the REC will be retained. It is the Chief Investigator’s responsibility to produce the annual reports as required. The Chief Investigator will notify the REC of the end of the study. An annual progress report (APR) will be submitted to the REC within 30 days of the anniversary date on which the favourable opinion was given, and annually until the study is declared ended. If the study is ended prematurely, the Chief Investigator will notify the REC, including the reasons for the premature termination. Within one year after the end of the study, the Chief Investigator will submit a final report with the results, including any publications/abstracts, to the REC.

Regulatory Review & Compliance

The Chief Investigator or designee will ensure that appropriate approvals from participating organisations are in place. Specific arrangements on how to gain approval from participating organisations are in place and comply with the relevant guidance. For any amendment to the study, the Chief Investigator or designee, in agreement with the sponsor will submit information to the appropriate body in order for them to issue approval for the amendment. The Chief Investigator or designee will ensure they can put the necessary arrangements in place to implement the amendment to confirm their support for the study as amended.

Amendments

The Chief Investigator will be responsible for the decision to amend the protocol and for deciding whether an amendment is substantial or non-substantial. The Chief Investigator will obtain advice from the sponsor, the HRA and/or REC in support of this decision. The Chief investigator will be responsible for communicating substantial changes to all relevant stakeholders including the sponsor, REC, HRA, and R&D. The amendment history will be tracked within the protocol and protocol number amended to correspond with this.

Patient & Public Involvement

Public involvement meetings will occur throughout the study alongside steering group meetings. Older adults have been involved directly in the design of study documents, including participant information sheets. Older adults will be invited to interim and final steering group meetings when considering preliminary and final analysis of results. An interim meeting will enable identification of any amendments to the study protocol that might be necessary, and a final meeting will assist with interpretation of the results, and planned dissemination.

Protocol compliance

Accidental protocol deviations can happen at any time. They must be adequately documented on the relevant forms and reported to the Chief Investigator and Sponsor immediately. Deviations from the protocol which are found to frequently recur are not acceptable, will require immediate action and could potentially be classified as a serious breach if the breach is assessed as likely to affect to a significant degree the safety or physical or mental integrity of the participants or the scientific value of the study. Events identified as serious breaches will be reported to the REC and sponsor in line with the Sponsor’s serious breaches reporting procedures. The Chief Investigator will be responsible for monitoring protocol compliance and allowing auditors access to study documentation as necessary.

Data protection and patient confidentiality

All investigators and study site staff must comply with the requirements of the Data Protection Act 2018 with regards to the collection, storage, processing, and disclosure of personal information and will uphold the Act’s core principles. Raw data will be collected on standardised paper case report forms (CRFs), which will be filed in the investigator site file (ISF). The ISF will be stored in a locked facility at The University of Birmingham. Data will be securely uploaded through REDCap, which is secure encrypted data management software. Access to REDCap will be provided to users at an appropriate level, with roles setup accordingly. Data access groups will be used to maintain separation of data upload and export across individual participating sites. Data will be archived for ten years.

Storage of Samples

The blood and adipose samples will be stored in -80 degree freezers in the Institute of Inflammation and Ageing, specifically the University of Birmingham Laboratories that sit within the Queen Elizabeth Hospital, Birmingham. This is only accessible via swipe access. Members of the research team with responsibility for laboratory based experiments or sample administration will have access. The freezers are temperature controlled with an in built alarm system to ensure the samples are kept at ambient temperature. The stool samples will be transferred to the Department of Twins Research at Kings College London. They will also be stored in -80 degree freezers with controlled access and in-built alarm systems.

The samples will be stored for a period of 10 years following the close of the study. The blood and adipose tissue will be stored at the University of Birmingham and the stool samples at the Department of Twins Research at Kings College London. This facility is HTA compliant.

The study steering committee will meet to determine the suitability of requests for samples for further research.

Indemnity

The University of Birmingham has in place indemnity coverage for this study which provides cover to the University for harm which comes about through the University’s, or its staff’s, negligence in relation to the design or management of the study and may alternatively, and at the University’s discretion provide cover for non-negligent harm to participants. The University of Birmingham is independent of any pharmaceutical company, and as such it is not covered by the Association of the British Pharmaceutical Industry guidelines for participant compensation.

With respect to the conduct of the study at Site and other clinical care of the patient, responsibility remains with the NHS organisation responsible for the clinical site and is therefore indemnified through NHS Resolution. The NHS have a duty of care to participants whether or not the participant is taking part in a clinical study.

End of study and archiving

The end of the study will be the date of the last visit of the last participant enrolled in this study. It will be the responsibility of the Chief Investigator to inform the REC that the study has ended within 90 days. The Principal Investigator will be responsible for archiving of data at site. Data will be stored for ten years in line with University of Birmingham guidelines.

Access to the final study dataset

The Chief Investigator and steering group will have direct access to the full dataset. The dataset will be fully anonymised upon study completion and the fully anonymised dataset will be available for further secondary research without the requirement for further ethical approval.

1. Dissemination

Dissemination policy

The data arising from the study will be the property of the investigators. Upon completion of the study, the data will be analysed and tabulated and a final study report prepared. The main study report and any sub-studies will be published in high impact peer-reviewed gerontology journals (e.g., Age and Ageing). All funding or supporting bodies will be acknowledged within publications, but they will not have reviewed and will not have publication rights of the data from the study.

The qualitative interview findings will form the basis of at least one dedicated manuscript describing older adults’ motivations for participation, perceptions of study procedures (for example adipose biopsy, stool sampling and EEG), tolerability of the interventions, and views on the future use of geroprotectors in clinical care. Qualitative insights will also be integrated into mixed-methods outputs where appropriate, to contextualise and interpret the biological and mechanistic findings. Key qualitative themes will be summarised in accessible language within participant result summaries and public engagement activities, with input from the PPI group to ensure clarity and relevance.

Participants enrolled in the study will be informed of the results by a written summary sent personally to them. The results will also be presented at academic conferences and through a series of public engagement events including webinars.

The study protocol may be submitted for publication within BMC Geriatrics or other peer-reviewed journal within the first year of ethical approval of the study. The anonymised participant level dataset will not be publicly available but will be available from the investigator upon reasonable request.

Authorship eligibility guidelines and any intended use of professional writers

The publications arising from this study will be authored by the writing group for each manuscript. The order of authorship may differ dependent on the publication. However, all protocol contributors, researchers at participating sites, and advisors will be acknowledged and listed as fully Pubmed-citable collaborative authors as members of the REPROGRAM Study Group. Professional writers will not be used.

1. References

1. Taylor JA, Greenhaff PL, Bartlett DB, Jackson TA, Duggal NA, Lord JM. Multisystem physiological perspective of human frailty and its modulation by physical activity. Physiol Rev. 2023;103(2):1137-91.

2. Berian JR, Mohanty S, Ko CY, Rosenthal RA, Robinson TN. Association of Loss of Independence With Readmission and Death After Discharge in Older Patients After Surgical Procedures. JAMA Surg. 2016;151(9):e161689.

3. Lopez-Otin C, Blasco MA, Partridge L, Serrano M, Kroemer G. Hallmarks of aging: An expanding universe. Cell. 2023;186(2):243-78.

4. Justice JN, Nambiar AM, Tchkonia T, LeBrasseur NK, Pascual R, Hashmi SK, et al. Senolytics in idiopathic pulmonary fibrosis: Results from a first-in-human, open-label, pilot study. EBioMedicine. 2019;40:554-63.

5. Trendelenburg AU, Scheuren AC, Potter P, Muller R, Bellantuono I. Geroprotectors: A role in the treatment of frailty. Mech Ageing Dev. 2019;180:11-20.

6. Bailey CJ. Metformin: historical overview. Diabetologia. 2017;60(9):1566-76.

7. Khan N, Syed DN, Ahmad N, Mukhtar H. Fisetin: a dietary antioxidant for health promotion. Antioxid Redox Signal. 2013;19(2):151-62.

8. Muñoz-Esparza NC, Latorre-Moratalla ML, Comas-Basté O, Toro-Funes N, Veciana-Nogués MT, Vidal-Carou MC. Polyamines in Food. Front Nutr. 2019;6.

9. Maher P. How fisetin reduces the impact of age and disease on CNS function. Front Biosci (Schol Ed). 2015;7(1):58-82.

10. Campbell JM, Bellman SM, Stephenson MD, Lisy K. Metformin reduces all-cause mortality and diseases of ageing independent of its effect on diabetes control: A systematic review and meta-analysis. Ageing Res Rev. 2017;40:31-44.

11. Wang CP, Lorenzo C, Habib SL, Jo B, Espinoza SE. Differential effects of metformin on age related comorbidities in older men with type 2 diabetes. J Diabetes Complications. 2017;31(4):679-86.

12. Martin-Montalvo A, Mercken EM, Mitchell SJ, Palacios HH, Mote PL, Scheibye-Knudsen M, et al. Metformin improves healthspan and lifespan in mice. Nat Commun. 2013;4.

13. Anisimov VN, Berstein LM, Egormin PA, Piskunova TS, Popovich IG, Zabezhinski MA, et al. Metformin slows down aging and extends life span of female SHR mice. Cell Cycle. 2008;7(17):2769-73.

14. Kulkarni AS, Gubbi S, Barzilai N. Benefits of Metformin in Attenuating the Hallmarks of Aging. Cell Metab. 2020;32(1):15-30.

15. Laksmi PW, Setiati S, Tamin TZ, Soewondo P, Rochmah W, Nafrialdi N, et al. Effect of Metformin on Handgrip Strength, Gait Speed, Myostatin Serum Level, and Health-related Quality of Life: A Double Blind Randomized Controlled among Non-diabetic Pre-frail Elderly Patients. Acta Med Indones. 2017;49(2):118-27.

16. Walton RG, Dungan CM, Long DE, Tuggle SC, Kosmac K, Peck BD, et al. Metformin blunts muscle hypertrophy in response to progressive resistance exercise training in older adults: A randomized, double-blind, placebo-controlled, multicenter trial: The MASTERS trial. Aging Cell. 2019;18(6):e13039.

17. Rennie KJ, Witham M, Bradley P, Clegg A, Connolly S, Hancock HC, et al. MET-PREVENT: metformin to improve physical performance in older people with sarcopenia and physical prefrailty/frailty - protocol for a double-blind, randomised controlled proof-of-concept trial. Bmj Open. 2022;12(7):e061823.

18. Barzilai N, Crandall JP, Kritchevsky SB, Espeland MA. Metformin as a Tool to Target Aging. Cell Metab. 2016;23(6):1060-5.

19. Elsallabi O, Patruno A, Pesce M, Cataldi A, Carradori S, Gallorini M. Fisetin as a Senotherapeutic Agent: Biopharmaceutical Properties and Crosstalk between Cell Senescence and Neuroprotection. Molecules. 2022;27(3).

20. Huard CA, Gao X, Dey Hazra ME, Dey Hazra RO, Lebsock K, Easley JT, et al. Effects of Fisetin Treatment on Cellular Senescence of Various Tissues and Organs of Old Sheep. Antioxidants (Basel). 2023;12(8).

21. Mullen M, Nelson AL, Goff A, Billings J, Kloser H, Huard C, et al. Fisetin Attenuates Cellular Senescence Accumulation During Culture Expansion of Human Adipose-Derived Stem Cells. Stem Cells. 2023;41(7):698-710.

22. Yousefzadeh MJ, Zhu Y, McGowan SJ, Angelini L, Fuhrmann-Stroissnigg H, Xu M, et al. Fisetin is a senotherapeutic that extends health and lifespan. EBioMedicine. 2018;36:18-28.

23. Chaib S, Tchkonia T, Kirkland JL. Cellular senescence and senolytics: the path to the clinic. Nat Med. 2022;28(8):1556-68.

24. Atiya Ali M, Poortvliet E, Stromberg R, Yngve A. Polyamines in foods: development of a food database. Food Nutr Res. 2011;55.

25. Hofer SJ, Simon AK, Bergmann M, Eisenberg T, Kroemer G, Madeo F. Mechanisms of spermidine-induced autophagy and geroprotection. Nat Aging. 2022;2(12):1112-29.

26. Kiechl S, Pechlaner R, Willeit P, Notdurfter M, Paulweber B, Willeit K, et al. Higher spermidine intake is linked to lower mortality: a prospective population-based study. Am J Clin Nutr. 2018;108(2):371-80.

27. Hickson LJ, Langhi Prata LGP, Bobart SA, Evans TK, Giorgadze N, Hashmi SK, et al. Senolytics decrease senescent cells in humans: Preliminary report from a clinical trial of Dasatinib plus Quercetin in individuals with diabetic kidney disease. EBioMedicine. 2019;47:446-56.

28. Ng M, Hazrati LN. Evidence of sex differences in cellular senescence. Neurobiol Aging. 2022;120:88-104.

29. Roberts HC, Denison HJ, Martin HJ, Patel HP, Syddall H, Cooper C, et al. A review of the measurement of grip strength in clinical and epidemiological studies: towards a standardised approach. Age and ageing. 2011;40(4):423-9.

30. Guralnik JM, Simonsick EM, Ferrucci L, Glynn RJ, Berkman LF, Blazer DG, et al. A short physical performance battery assessing lower extremity function: association with self-reported disability and prediction of mortality and nursing home admission. Journal of gerontology. 1994;49(2):M85-94.

31. Mahoney FI, Barthel DW. Functional evaluation: The Barthel Index: A simple index of independence useful in scoring improvement in the rehabilitation of the chronically ill. Maryland State Medical Journal. 1965;14:61-5.

32. Nouri F, Lincoln N. An extended activities of daily living scale for stroke patients. Clinical rehabilitation. 1987;1(4):301-5.

33. Nasreddine ZS, Phillips NA, Bédirian V, Charbonneau S, Whitehead V, Collin I, et al. The Montreal Cognitive Assessment, MoCA: a brief screening tool for mild cognitive impairment. Journal of the American Geriatrics Society. 2005;53(4):695-9.

34. Fan J, McCandliss BD, Sommer T, Raz A, Posner MI. Testing the efficiency and independence of attentional networks. Journal of cognitive neuroscience. 2002;14(3):340-7.

35. Markiewicz R, Segaert K, Mazaheri A. How the healthy ageing brain supports semantic binding during language comprehension. European Journal of Neuroscience. 2021;54(11):7899-917.

36. Segaert K, Poulisse C, Markiewicz R, Wheeldon L, Marchment D, Adler Z, et al. Detecting impaired language processing in patients with mild cognitive impairment using around-the-ear cEEgrid electrodes. Psychophysiology. 2022;59(5):e13964.

37. Strasser EM, Draskovits T, Praschak M, Quittan M, Graf A. Association between ultrasound measurements of muscle thickness, pennation angle, echogenicity and skeletal muscle strength in the elderly. Age (Dordrecht, Netherlands). 2013;35(6):2377-88.

38. Wilson D. Frailty, sarcopenia and immunesenescence: shared mechanisms and clinical insights. University of Birmingham: University of Birmingham; 2018.

39. Perkisas S, Baudry S, Bauer J, Beckwée D, De Cock A-M, Hobbelen H, et al. Application of ultrasound for muscle assessment in sarcopenia: towards standardized measurements. European Geriatric Medicine. 2018.

40. Karimi M, Brazier J. Health, Health-Related Quality of Life, and Quality of Life: What is the Difference? Pharmacoeconomics. 2016;34(7):645-9.

41. Berian JR, Mohanty S, Ko CY, Rosenthal RA, Robinson TN. Association of Loss of Independence With Readmission and Death After Discharge in Older Patients After Surgical Procedures. JAMA Surgery. 2016;151(9):e161689-e.

42. Saul D, Kosinsky RL, Atkinson EJ, Doolittle ML, Zhang X, LeBrasseur NK, et al. A new gene set identifies senescent cells and predicts senescence-associated pathways across tissues. Nature Communications. 2022;13(1):4827.

43. George EL, Hall DE, Youk A, Chen R, Kashikar A, Trickey AW, et al. Association Between Patient Frailty and Postoperative Mortality Across Multiple Noncardiac Surgical Specialties. JAMA Surgery. 2021;156(1):e205152-e.

44. Yousefzadeh MJ, Flores RR, Zhu Y, Schmiechen ZC, Brooks RW, Trussoni CE, et al. An aged immune system drives senescence and ageing of solid organs. Nature. 2021;594(7861):100-5.

45. Foster MA, Bentley C, Hazeldine J, Acharjee A, Nahman O, Shen-Orr SS, et al. Investigating the potential of a prematurely aged immune phenotype in severely injured patients as predictor of risk of sepsis. Immunity & Ageing. 2022;19(1):60.

46. Yoshii SR, Mizushima N. Monitoring and Measuring Autophagy. Int J Mol Sci. 2017;18(9).

47. Hamann PD, Roux BT, Heward JA, Love S, McHugh NJ, Jones SW, et al. Transcriptional profiling identifies differential expression of long non-coding RNAs in Jo-1 associated and inclusion body myositis. Scientific Reports. 2017;7(1):8024.

48. Cruz-Jentoft AJ, Bahat G, Bauer J, Boirie Y, Bruyère O, Cederholm T, et al. Sarcopenia: revised European consensus on definition and diagnosis. Age and ageing. 2019;48(1):16-31.

49. Wilson DV, Moorey H, Stringer H, Sahbudin I, Filer A, Lord JM, et al. Bilateral Anterior Thigh Thickness: A New Diagnostic Tool for the Identification of Low Muscle Mass? Journal of the American Medical Directors Association. 2019;20(10):1247-53.e2.

50. Fried LP, Tangen CM, Walston J, Newman AB, Hirsch C, Gottdiener J, et al. Frailty in older adults: evidence for a phenotype. The journals of gerontology Series A, Biological sciences and medical sciences. 2001;56(3):M146-56.

51. Romero-Ortuno R, Walsh CD, Lawlor BA, Kenny RA. A Frailty Instrument for primary care: findings from the Survey of Health, Ageing and Retirement in Europe (SHARE). BMC Geriatrics. 2010;10(1):57.

52. National Institute for Health Research. Improving inclusion of under-served groups in clinical research: guidance from the NIHR INCLUDE project. London: NIHR; 2020.()

53. National Institute for Health and Care Research. Be Part of Research [Internet]. London: NIHR; 2023. Available from: https://bepartofresearch.nihr.ac.uk/ ()

54. McGrath C, Palmgren PJ, Liljedahl M. Twelve tips for conducting qualitative research interviews. Med Teach. 2019;41(9):1002–1006.()

55. Ritchie J, Spencer L. Qualitative data analysis for applied policy research. In: Bryman A, Burgess R, editors. Analysing qualitative data. London: Routledge; 1994. p.173–194.()

56. Michie S, van Stralen MM, West R. The behaviour change wheel: a new method for characterising and designing behaviour change interventions. Implement Sci. 2011;6:42.()

57. Malterud K, Siersma VD, Guassora AD. Sample size in qualitative interview studies: guided by information power. Qual Health Res. 2016;26(13):1753–1760.()

58. Braun V, Clarke V. Using thematic analysis in psychology. Qual Res Psychol. 2006;3(2):77–101.()

59. Skivington K, Matthews L, Simpson SA, Craig P, Baird J, Blazeby JM, et al. A new framework for developing and evaluating complex interventions: update of Medical Research Council guidance. BMJ. 2021;374.()

Appendix

Criteria for sarcopenia diagnosis

Cut-off values used for sarcopenia diagnosis. Cut-off values for handgrip strength, SMMSergi, gait speed, and SPPB are taken from those recommended by the European Working Group in Older People 2 (48). Cut-off values for BATT are taken from those recommended by Wilson et al (49).

*BATT=Bilateral Anterior Thigh Thickness; SMMSergi=Skeletal Muscle Mass (Sergi equation)*.

|  | **Male** | **Female** |
| --- | --- | --- |
| No sarcopenia | 1. Handgrip strength ≥27kg | 1. Handgrip strength ≥16kg |
| Probable sarcopenia | 1. Handgrip strength <27kg | 1. Handgrip strength <16kg |
|  | 2. BATT ≥5.44cm AND SMMSergi ≥20kg | 2. BATT ≥3.85cm AND SMMSergi ≥20kg |
| Definite sarcopenia, not severe | 1. Handgrip strength <27kg | 1. Handgrip strength <16kg |
|  | 2. BATT <5.44cm AND/OR SMMSergi <20kg | 2. BATT <3.85cm AND/OR SMMSergi <15kg |
|  | 3. Gait speed >0.8m/s AND SPPB >8 | 3. Gait speed >0.8m/s AND SPPB >8 |
| Definite sarcopenia, severity unclear | 1. Handgrip strength <27kg | 1. Handgrip strength <16kg |
|  | 2. BATT <5.44cm AND/OR SMMSergi <20kg | 2. BATT <3.85cm AND/OR SMMSergi <15kg |
| 3. Gait speed not measured AND SPPB not measured | 3. Gait speed not measured AND SPPB not measured | |
| Definite sarcopenia, severe | 1. Handgrip strength <27kg | 1. Handgrip strength <16kg |
|  | 2. BATT <5.44cm AND/OR SMMSergi <20kg | 2. BATT <3.85cm AND/OR SMMSergi <15kg |
|  | 3. Gait speed ≤0.8m/s AND SPPB ≤8 | 3. Gait speed ≤0.8m/s AND SPPB ≤8 |

Short Physical Performance Battery (SPPB)

|  | Raw times (sec) | Time scoring | Score (circle) | If did not attempt or unable*: |
| --- | --- | --- | --- | --- |
| Side-by-side stand | __ __ : __ __ | Not attempted or not held for 10 sec | 0 |  |
|  |  | Held for 10 sec | 1 |  |
| Semi-tandem stand | __ __ : __ __ | Not attempted or not held for 10 sec | 0 |  |
|  |  | Held for 10 sec | 1 |  |
| Tandem stand | __ __ : __ __ | Not attempted or not held for 3 sec | 0 |  |
|  |  | Held for 3 – 9.99 sec | 1 |  |
|  |  | Held for 10 sec | 2 |  |
| Gait speed test (4m walk) | __ __ : __ __ | Unable | 0 |  |
|  |  | > 8.70 sec | 1 |  |
|  |  | 6.21 – 8.70 sec | 2 |  |
|  |  | 4.82 – 6.20 sec | 3 |  |
|  |  | < 4.82 sec | 4 |  |
| 5 chair stands | __ __ : __ __ | Unable to complete chair stands or takes > 60 sec | 0 |  |
|  |  | >= 16.70 sec | 1 |  |
|  |  | 13.70 – 16.69 sec | 2 |  |
|  |  | 11.20 – 13.69 sec | 3 |  |
|  |  | <= 11.19 sec | 4 |  |

Aids for gait speed test? None ❒ Walking stick ❒Zimmer frame ❒Other ❒

*If patient did not attempt test or failed, use the following key:

Tried but unable 1

Participant could not hold position/ stand/ walk unassisted 2

Not attempted, investigator felt unsafe 3

Not attempted, participant felt unsafe 4

Participant unable to understand instructions 5

Other (specify) 6

Participant refused 7

Barthel Index

Barthel Index

Please circle the most appropriate responses

|  | 0 | 1 | 2 | 3 |
| --- | --- | --- | --- | --- |
| Bowels | Incontinent | Occasional accident | Continent |  |
| Bladder | Incontinent (or catheter unable to manage independently) | Occasional accident (max once per 24 hours) | Continent (over 7-day period) |  |
| Grooming | Needs help with personal care | Independent face/hair/teeth/shaving |  |  |
| Toilet Use | Dependent | Needs some help | Independent |  |
| Feeding | Unable | Needs help e.g. cutting | Independent |  |
| Transfers | Unable – no sitting balance | Major help but can sit | Minor help (physical or verbal) | Independent |
| Mobility | Immobile | Wheelchair independent | Walks with help of one person | Independent (may use aids) |
| Dressing | Dependent | Needs help, can do about half | Independent |  |
| Stairs | Unable | Needs help | Independent |  |
| Bathing | Dependent | Independent |  |  |

Barthel Index Score _________

Nottingham Extended Activities of Daily Living

Nottingham Extended Activities of Daily Living

This should be completed according to the activities that the participant has actually done over the last four weeks. Please tick the most appropriate responses.

|  | 0 | | 1 | |
| --- | --- | --- | --- | --- |
|  | Not at all | With help | On own with difficulty | On own |
| Walked around outside? |  |  |  |  |
| Climbed stairs? |  |  |  |  |
| Gotten in and out of car? |  |  |  |  |
| Walked over uneven ground? |  |  |  |  |
| Crossed roads? |  |  |  |  |
| Travelled on public transport? |  |  |  |  |
| Managed to feed yourself? |  |  |  |  |
| Made a hot drink? |  |  |  |  |
| Taken hot drinks from one room to another? |  |  |  |  |
| Done the washing up? |  |  |  |  |
| Made a hot snack? |  |  |  |  |
| Managed your own money? |  |  |  |  |
| Washed small items of clothing? |  |  |  |  |
| Done your own housework? |  |  |  |  |
| Done your own shopping? |  |  |  |  |
| Done a full clothes wash? |  |  |  |  |
| Read newspapers or books? |  |  |  |  |
| Used the telephone? |  |  |  |  |
| Written letters? *(or emails)* |  |  |  |  |
| Gone out socially? |  |  |  |  |
| Managed your own garden? |  |  |  |  |
| Driven a car? |  |  |  |  |

NEADL Score _______

Criteria used to make a phenotypic diagnosis of frailty

| Criteria | Criteria to score positive | | Source |
| --- | --- | --- | --- |
| Handgrip strength (kg) | Male | Female | Original study (50) |
|  | BMI <= 24: <=29  BMI 24-26: <=30  BMI 26-28: <=30  BMI >28: <=32 | BMI <=24: <=17  BMI 24-26: <=17.3  BMI 26-28: <=18  BMI >28: <=21 |  |
| Gait speed (m/s) | Male | Female | Original study (50) |
|  | <=1.73m height: <=0.65  >1.73m height: <=0.76 | <= 1.59m height: <= 0.65  >1.59m height: <=0.76 |  |
| Self-reported exhaustion | Answers “most of the time” or “all of the time” to the following questions.  How often over the last week have you felt that the following statements were true:   - “I felt that everything I did was an effort” - “I could not get getting” | | Original study (50) |
| Weight loss | 4.5kg OR 5% total body weight loss over last year | | Original study (50) |
| Physical activity | How often do you engage in activities that require a low or moderate level of energy such as gardening, cleaning the car, or doing a walk?   - More than once a week - Once a week - One to three times a month - Hardly ever or never | | SHARE-FI (51) |

Scoring criteria for CFS

| CFS score | Description |
| --- | --- |
| 1  Very fit | Robust, active, energetic and motivated; commonly exercise regularly |
| 2  Fit | No active disease symptoms but less fit than above; exercise often or active occasionally e.g. seasonally |
| 3  Managing well | Medical problems are well controlled but not regularly active beyond routine walking |
| 4  Living with very mild frailty | Not dependent on others for daily help but often symptoms limit activities; common complaint is being “slowed-up” or tired during the day |
| 5  Living with mild frailty | More evident slowing; need help with high order IADLs; typically progressively impairs shopping and walking outside alone, meal preparation and housework |
| 6  Living with moderate frailty | Need help with all outside activities; often have problems with stairs and need help with bathing and might need minimal assistance with dressing |
| 7  Living with severe frailty | Completely dependent for personal care; seem stable and not at high risk of dying within 6 months |
| 8  Living with very severe frailty | Completely dependent, approaching the end of life; typically, could not recover from a minor illness |
| 9  Terminally ill | Life expectancy < 6 months but not otherwise evidently frail |

Frailty Index

| Deficit | Definition |
| --- | --- |
| Activity limitation | Positive Fried physical activity score |
| Anaemia and haematinic deficiency | Female Hb<115, Male Hb<135, on medication for haematinic deficiency, or new haematinic deficiency identified during admission |
| Arthritis | Patient reported (includes osteoarthritis and inflammatory arthritis) |
| Atrial fibrillation | Any history – paroxysmal, temporary, or permanent |
| Cerebrovascular disease | Vascular dementia or stroke disease |
| Chronic kidney disease | eGFR <60 |
| Diabetes mellitus | Known history/ confirmed diagnosis |
| Dizziness | Patient reported |
| Dyspnoea | Patient reported |
| Falls | Two or more over previous year |
| Foot problems | Patient reported |
| Fragility fracture | Previous history |
| Hearing impairment | Need for hearing aids |
| Heart failure | Known history/ confirmed diagnosis |
| Heart valve disease | Known history |
| Housebound | Nottingham extended ADLs |
| Hypertension | On treatment or recorded |
| Presyncope/ syncope | Patient reported |
| Ischaemic heart disease | Known history |
| Memory and cognitive problems | Any cognitive spectrum disorder including mild cognitive impairment, delirium, and dementia |
| Osteoporosis | On treatment or known history |
| Parkinsonism and tremor | Includes tremor of any cause – known history or on treatment |
| Peptic ulcer | Known history |
| Peripheral vascular disease | Known history |
| Polypharmacy | ≥5 prescribed medications |
| Requirement for care | Formal carers |
| Respiratory disease | Any history of chronic disease e.g. asthma, COPD |
| Skin ulcer | Any current skin ulcer including arterial, venous, or pressure-related |
| Sleep disturbance | Patient reported |
| Social vulnerability | Lives alone |
| Thyroid disease | Known history |
| Urinary or faecal incontinence | Barthel index |
| Urinary system disease | Known history |
| Visual impairment | Wears glasses/ visual aids or on treatment for eye condition(s) |
| Weight loss and anorexia | Fried weight loss |
