# Supplementary material for: REPROGRAM: REsilience PROmotion with GeRoprotectors: AssessMent of biological effect: Rationale and protocol for a trial of biological effect
Source: PLoS One. 2026 Jun 17;21(6):e0346347. doi: 10.1371/journal.pone.0346347 (PMC13274875; doi:10.1371/journal.pone.0346347)
Supplement: S3 File — (DOCX) [file pone.0346347.s003.docx]

# Supplementary Materials

## Side Effect Profile of Agents

Metformin – as listed in the British National Formulary (BNF)(1)

**Common or very common**

Abdominal pain; appetite decreased; diarrhoea; gastrointestinal disorder; nausea; taste altered; vitamin B12 deficiency; vomiting

**Rare or very rare**

Hepatitis; lactic acidosis (discontinue); skin reactions

**Side-effects, further information**

Gastrointestinal side-effects are most frequent during treatment initiation and usually resolve spontaneously. A slow increase in dose may improve tolerability.

Fisetin

The data on side effects for nutraceuticals is not as robust for drugs. Reported side effects are minimal but related to gastric discomfort (2, 3). Animal models have reported no toxicity up to 2000mg/kg in mice and no long-term toxicity or mutagenicity (4). Caution is advised when taken concurrently with drugs metabolised by the cytochrome P450 family as it can inhibit these enzymes.

Spermidine

The data on side effects for nutraceuticals is not as robust for drugs. Of note spermidine is derived from wheat and therefore we have excluded participants with coealiac disease from receiving spermidine. Reported side effects are minimal but related to gastric discomfort (5-7). Toxicity studies have been conducted in rats which demonstrate very high doses are required before toxicity is reached (8).

## Geroprotector Suppliers and Preparations

Metformin – Supplied by NHS Pharmacy. 1500mg MR preparation.

Spermidine – Supplied by Longevity Box. Custom preparation. 15mg wheat-derived spermidine. Independent assessment of Spermidine using mass HLPC analysis confirmed >97% active ingredient.

Fisetin – Supplied by Longevity Box. Custom preparation. 100mg. Independent assessment of Fisetin using HLPC analysis confirmed >97% active ingredient.

## Frailty Index

| **Deficit** | **Definition** |
| --- | --- |
| Activity limitation | Positive Fried physical activity score |
| Anaemia and haematinic deficiency | Female Hb<115, Male Hb<135, on medication for haematinic deficiency, or new haematinic deficiency identified during admission |
| Arthritis | Patient reported (includes osteoarthritis and inflammatory arthritis) |
| Atrial fibrillation | Any history – paroxysmal, temporary, or permanent |
| Cerebrovascular disease | Vascular dementia or stroke disease |
| Chronic kidney disease | eGFR <60 |
| Diabetes mellitus | Known history/ confirmed diagnosis |
| Dizziness | Patient reported |
| Dyspnoea | Patient reported |
| Falls | Two or more over previous year |
| Foot problems | Patient reported |
| Fragility fracture | Previous history |
| Hearing impairment | Need for hearing aids |
| Heart failure | Known history/ confirmed diagnosis |
| Heart valve disease | Known history |
| Housebound | Nottingham extended ADLs |
| Hypertension | On treatment or recorded |
| Presyncope/ syncope | Patient reported |
| Ischaemic heart disease | Known history |
| Memory and cognitive problems | Any cognitive spectrum disorder including mild cognitive impairment, delirium, and dementia |
| Osteoporosis | On treatment or known history |
| Parkinsonism and tremor | Includes tremor of any cause – known history or on treatment |
| Peptic ulcer | Known history |
| Peripheral vascular disease | Known history |
| Polypharmacy | ≥5 prescribed medications |
| Requirement for care | Formal carers |
| Respiratory disease | Any history of chronic disease e.g. asthma, COPD |
| Skin ulcer | Any current skin ulcer including arterial, venous, or pressure-related |
| Sleep disturbance | Patient reported |
| Social vulnerability | Lives alone |
| Thyroid disease | Known history |
| Urinary or faecal incontinence | Barthel index |
| Urinary system disease | Known history |
| Visual impairment | Wears glasses/ visual aids or on treatment for eye condition(s) |
| Weight loss and anorexia | Fried weight loss |

## Frailty Phenotype

| Criteria | Criteria to score positive | | Source |
| --- | --- | --- | --- |
| Handgrip strength (kg) | Male | Female | Original study (9) |
|  | BMI <= 24: <=29  BMI 24-26: <=30  BMI 26-28: <=30  BMI >28: <=32 | BMI <=24: <=17  BMI 24-26: <=17.3  BMI 26-28: <=18  BMI >28: <=21 |  |
| Gait speed (m/s) | Male | Female | Original study (9) |
|  | <=1.73m height: <=0.65  >1.73m height: <=0.76 | <= 1.59m height: <= 0.65  >1.59m height: <=0.76 |  |
| Self-reported exhaustion | Answers “most of the time” or “all of the time” to the following questions.  How often over the last week have you felt that the following statements were true:   - “I felt that everything I did was an effort” - “I could not get getting” | | Original study (9) |
| Weight loss | 4.5kg OR 5% total body weight loss over last year | | Original study (9) |
| Physical activity | How often do you engage in activities that require a low or moderate level of energy such as gardening, cleaning the car, or doing a walk?   - More than once a week - Once a week - One to three times a month - Hardly ever or never | | SHARE-FI (10) |

## Sarcopenia Diagnosis

Cut-off values used for sarcopenia diagnosis. Cut-off values for handgrip strength, SMMSergi, gait speed, and SPPB are taken from those recommended by the European Working Group in Older People 2 (11). Cut-off values for BATT are taken from those recommended by Wilson et al (12).

*BATT=Bilateral Anterior Thigh Thickness; SMMSergi=Skeletal Muscle Mass (Sergi equation)*.

|  | **Male** | **Female** |
| --- | --- | --- |
| No sarcopenia | 1. Handgrip strength ≥27kg | 1. Handgrip strength ≥16kg |
| Probable sarcopenia | 1. Handgrip strength <27kg | 1. Handgrip strength <16kg |
|  | 2. BATT ≥5.44cm | 2. BATT ≥3.85cm |
| Definite sarcopenia, not severe | 1. Handgrip strength <27kg | 1. Handgrip strength <16kg |
|  | 2. BATT <5.44cm | 2. BATT <3.85cm |
|  | 3. Gait speed >0.8m/s | 3. Gait speed >0.8m/s |
| Severe sarcopenia | 1. Handgrip strength <27kg | 1. Handgrip strength <16kg |
|  | 2. BATT <5.44cm | 2. BATT <3.85cm |
|  | 3. Gait speed ≤ 0.8m/s | 3. Gait speed ≤ 0.8m/s |

1. Committee JF. British National Formulary [Internet]. BMJ Group and Pharmaceutical Press; 2025.

2. Verdoorn BP, Evans TK, Hanson GJ, Zhu Y, Langhi Prata LGP, Pignolo RJ, et al. Fisetin for COVID-19 in skilled nursing facilities: Senolytic trials in the COVID era. J Am Geriatr Soc. 2021;69(11):3023-33.

3. Hodgin KS, Donovan EK, Kekes-Szabo S, Lin JC, Feick J, Massey RL, et al. A Placebo-Controlled, Pseudo-Randomized, Crossover Trial of Botanical Agents for Gulf War Illness: Resveratrol (Polygonum cuspidatum), Luteolin, and Fisetin (Rhus succedanea). Int J Environ Res Public Health. 2021;18(5).

4. Seal I, Sil S, Das A, Roy S. Assessment of toxicity and genotoxic safety profile of novel fisetin ruthenium-p-cymene complex in mice. Toxicol Res. 2023;39(2):213-29.

5. Schwarz C, Stekovic S, Wirth M, Benson G, Royer P, Sigrist SJ, et al. Safety and tolerability of spermidine supplementation in mice and older adults with subjective cognitive decline. Aging (Albany NY). 2018;10(1):19-33.

6. Keohane P, Everett JR, Pereira R, Cook CM, Blonquist TM, Mah E. Supplementation of spermidine at 40 mg/day has minimal effects on circulating polyamines: An exploratory double-blind randomized controlled trial in older men. Nutr Res. 2024;132:1-14.

7. Schwarz C, Benson GS, Horn N, Wurdack K, Grittner U, Schilling R, et al. Effects of Spermidine Supplementation on Cognition and Biomarkers in Older Adults With Subjective Cognitive Decline: A Randomized Clinical Trial. JAMA Netw Open. 2022;5(5):e2213875.

8. Chrysostomou PP, Freeman EL, Murphy MM, Pereira R, Esdaile DJ, Keohane P. A toxicological assessment of spermidine trihydrochloride produced using an engineered strain of Saccharomyces cerevisiae. Food Chem Toxicol. 2024;184:114428.

9. Fried LP, Tangen CM, Walston J, Newman AB, Hirsch C, Gottdiener J, et al. Frailty in older adults: evidence for a phenotype. J Gerontol A Biol Sci Med Sci. 2001;56(3):M146-56.

10. Romero-Ortuno R, Walsh CD, Lawlor BA, Kenny RA. A Frailty Instrument for primary care: findings from the Survey of Health, Ageing and Retirement in Europe (SHARE). BMC Geriatrics. 2010;10(1):57.

11. Cruz-Jentoft AJ, Bahat G, Bauer J, Boirie Y, Bruyère O, Cederholm T, et al. Sarcopenia: revised European consensus on definition and diagnosis. Age and ageing. 2019;48(1):16-31.

12. Wilson DV, Moorey H, Stringer H, Sahbudin I, Filer A, Lord JM, et al. Bilateral Anterior Thigh Thickness: A New Diagnostic Tool for the Identification of Low Muscle Mass? Journal of the American Medical Directors Association. 2019;20(10):1247-53.e2.
